# Supplementary material for: Multicentre randomised controlled trial: protocol for Plasma-Lyte Usage and Assessment of Kidney Transplant Outcomes in Children (PLUTO)
Source: BMJ Open. 2022 Mar 13;12(3):e055595. doi: 10.1136/bmjopen-2021-055595 (PMC8921856; doi:10.1136/bmjopen-2021-055595)
Supplement: Author's manuscript [file bmjopen-2021-055595.draft_revisions.pdf]

BMJ Open

BMJ Open is committed to open peer review. As part of this commitment we make the peer review history of every article we publish publicly available.

When an article is published we post the peer reviewers' comments and the authors' responses online. We also post the versions of the paper that were used during peer review. These are the versions that the peer review comments apply to.

The versions of the paper that follow are the versions that were submitted during the peer review process. They are not the versions of record or the final published versions. They should not be cited or distributed as the published version of this manuscript.

BMJ Open is an open access journal and the full, final, typeset and author-corrected version of record of the manuscript is available on our site with no access controls, subscription charges or pay-per-view fees (http://bmjopen.bmj.com).

If you have any questions on BMJ Open's open peer review process please email info.bmjopen@bmj.com

BMJ Open

Protocol for multi-centre randomised controlled trial: Plasma-Lyte Usage and assessment of kidney Transplant Outcomes in children (PLUTO)

Journal:	*BMJ Open*
Manuscript ID	bmjopen-2021-055595
Article Type:	Protocol
Date Submitted by the Author:	19-Jul-2021
Complete List of Authors:	Hayes, Wesley; Great Ormond Street Hospital for Children NHS Foundation Trust; University College London<br>Laing, Emma; NHS Blood and Transplant, Clinical Trials Unit<br>Foley, Claire; NHSBT Clinical Trials Unit<br>Pankhurst, Laura; NHS Blood and Transplant<br>Thomas, Helen; NHSBT Clinical Trials Unit<br>Hume-Smith, Helen; Great Ormond Street Hospital for Children NHS Foundation Trust; University College London<br>Marks, Dr. Stephen; Great Ormond Street Hospital For Children NHS Trust, Paediatric Nephrology<br>Kessaris, Nicos; Guy's Hospital, Department of Nephrology and Transplantation<br>Bryant, William A.; Great Ormond Street Hospital for Children NHS Foundation Trust<br>Spiridou, Anastassia; Great Ormond Street Hospital for Children NHS Foundation Trust<br>Wray, Jo; Great Ormond Street Hospital for Children, Heart and Lung Directorate<br>Peters, Mark; Great Ormond Street Hospital For Children NHS Trust, Paediatric Intensive Care Unit; University College London, Institute of Child Health
Keywords:	Renal transplantation < NEPHROLOGY, Paediatric nephrology < NEPHROLOGY, TRANSPLANT MEDICINE

SCHOLARONE™
Manuscripts

Protocol for multi-centre randomised controlled trial: Plasma-Lyte Usage and assessment of kidney Transplant Outcomes in children (PLUTO)

Wesley N Hayes[1,2], Emma Laing[3], Claire Foley[3], Laura Pankhurst[3], Helen Thomas[3], Helen Hume-Smith[1,2], Stephen D Marks[1,2], Nicos Kessaris[1,4], William Bryant[1], Anastassia Spiridou[1], Jo Wray[1], Mark J Peters[2,1]

1. Great Ormond Street Hospital for Children NHS Foundation Trust, and NIHR Biomedical Research Centre, WC1N 3JH, London, United Kingdom
2. University College London, Great Ormond Street Institute for Child Health, London, UK
3. National Health Service Blood and Transplant Clinical Trials Unit, UK
4. Guys and St Thomas NHS Foundation Trust, London UK

Corresponding Author:

Wesley N Hayes, Wesley.Hayes@gosh.nhs.uk

ORCID-ID:

Wesley Hayes (ORCID 0000-0002-8091-6945)

Stephen D Marks (ORCID 0000-0001-9850-8352)

Emma Laing (ORCID 0000-0002-8309-0990)

Anastassia Spiridou (ORCID 0000-0001-6576-0244)

Mark J Peters (ORCID 0000-0003-3653-4808)

For peer review only

Abstract

Introduction

Acute electrolyte and acid-base imbalance is experienced by many children following kidney transplantation. When severe this can lead to complications including seizures, cerebral oedema and death. Relatively large volumes of intravenous fluid are administered to children perioperatively in order to establish perfusion to the donor kidney. Hypotonic intravenous fluid is commonly used due to clinicians' concerns about the sodium, chloride and potassium content of isotonic alternatives when administered in large volumes.

Plasma-Lyte-148 is an isotonic, balanced intravenous fluid. There is a physiological basis to expect that Plasma-Lyte-148 will reduce the incidence of clinically significant electrolyte and acid-base abnormalities in children following kidney transplantation.

The aim of the PLUTO trial is to determine whether the incidence of clinically significantly abnormal plasma electrolyte levels in paediatric kidney transplant recipients will be different with the use of Plasma-Lyte-148 compared to intravenous fluid currently administered.

Methods and analysis

PLUTO is a pragmatic, open-label, randomised controlled trial comparing Plasma-Lyte 148 to current care in paediatric kidney transplant recipients, conducted in 9 UK centres.

A total of 144 children receiving kidney transplants will be randomised to receive either PlasmaLyte-148 (the intervention) intra- and postoperatively, or current fluid. Apart from intravenous fluid composition, all participants will receive standard clinical transplant care.

The primary outcome measure is acute hyponatraemia in the first 72 hours post-transplant. Secondary outcomes include symptoms of acute hyponatraemia, other electrolyte and acid base imbalances and transplant kidney function.

The primary outcome will be analysed using a logistic regression model adjusting for donor type, patient weight and transplant centre.

Ethics and dissemination

The trial has received Health Research Authority approval. Findings will be presented to academic groups via conferences and peer-reviewed journals. The patient and public involvement group will play an important part in disseminating the study findings into the public domain.

Registration details

EudraCT Number: 2019-003025-22

ISRCTN: 16586164

For peer review only

Strengths and Limitations of this study

- This is the first randomised controlled trial to evaluate balanced isotonic intravenous fluid in paediatric kidney transplant recipients
- Outcomes will provide evidence for safe perioperative fluid management in children undergoing kidney transplantation
- A process evaluation will be used to tailor the trial to optimise children and families' experience of participating
- Variation in current clinical practice between centres and clinicians makes standardisation of fluid administration in the control arm impractical
- Blinding was not considered necessary due to the objective nature of outcome measures

For peer review only

Introduction

Kidney transplantation is the treatment of choice for children with end stage kidney disease(1). Currently, acute electrolyte and acid-base imbalance is experienced by many paediatric recipients in the post-operative period(2). Abnormalities include acute hyponatraemia, hyperkalaemia and metabolic acidosis; smaller recipients of deceased donor kidneys are most at risk(2).

Acute hyponatraemia is closely associated with clinical complications in children after transplant: cerebral oedema, seizures(3) and death(4). Up to 8% of children suffer hyponatraemia-related seizures following kidney transplant(3). Pre-pubertal children are at particular risk of complications from acute hyponatraemia because of the relatively low ratio of cerebrospinal fluid and brain volume compared to older children and adults(5, 6). Acute hyponatraemia also predicts symptoms such as nausea and vomiting, headaches, breathlessness and reduced conscious level(7, 8).

Several factors predispose children to acute hyponatraemia following kidney transplant:

Firstly, relatively large volumes of intravenous fluid are administered perioperatively in order to establish and maintain perfusion to the adult donor kidney(9). Secondly, hypotonic intravenous fluid is predominantly administered postoperatively in paediatric kidney transplant centres(2). Thirdly, the post-operative period is characterised by an acute antidiuretic response which limits water excretion(8, 10).

Children receiving isotonic fluid as opposed to hypotonic fluid for indications other than kidney transplant experience less hyponatraemia(11-15). A randomised trial in children with sepsis comparing balanced fluid to 0.9% sodium chloride is currently underway (Pragmatic Pediatric Trial of Balanced vs Normal Saline Fluid in Sepsis (PRoMPT BOLUS) study, Clinicaltrials.gov NCT03340805)(16). National guidance now recommends isotonic fluid for children in the general paediatric setting(17). However, this guidance has not been adopted in paediatric transplant care because of a view that the period following kidney transplantation differs from other situations in terms of the volume of fluid administered and the associated sodium and chloride load. Hence, hypotonic fluid is still routinely administered to children following kidney transplantation(2, 3) and the use of isotonic fluid in this context requires evaluation.

Plasma-Lyte-148 is an isotonic, balanced intravenous fluid(18). It is licenced in children and contains sodium, chloride, potassium, magnesium with concentrations equivalent to those of plasma. There is a physiological basis to expect that Plasma-Lyte-148 will reduce the incidence of clinically significant electrolyte and acid-base abnormalities in children following kidney transplantation compared to current standard fluid.

A perceived risk of hyperkalaemia from the potassium content of Plasma-Lyte concerns some paediatric nephrologists, although a systematic review in adult kidney transplant recipients demonstrated less hyperkalaemia with Plasma-Lyte compared to 0.9% sodium chloride(19). A randomised trial(20) and a retrospective analysis in adult kidney transplant recipients(21) found a lower risk of hyperkalaemia with Plasma-Lyte-148 compared to 0.9% sodium chloride.

Currently, there is equipoise regarding use of balanced isotonic fluid vs hypotonic and chloride rich solutions for children undergoing kidney transplant.

The aim of the PLUTO trial is to determine whether the incidence of abnormal and potentially dangerous plasma electrolyte levels in paediatric kidney transplant recipients will be different with the use of Plasma-Lyte-148 compared to current standard intravenous fluids.

For peer review only

Methods and Analysis

Study design and setting

PLUTO is an open-label randomised controlled trial comparing Plasma-Lyte 148 (the intervention) to current intravenous fluids in paediatric kidney transplant recipients, conducted in 9 UK paediatric kidney transplant centres plus 3 participant identification centres (Table 1).

Intervention and comparator

Plasma-Lyte® 148 solution for infusion and Plasma-Lyte® 148 & Glucose 5% w/v solution for infusion are licenced in children and used in paediatric intensive care, surgical, and general paediatric wards. They contain sodium 140mmol/l, chloride 98mmol/l, potassium 5mmol/l, magnesium 1.5mmol/l and gluconate/acetate buffer to maintain physiological pH(18). Both solutions can be administered interchangeably within the intervention arm.

The comparator is the current paediatric kidney transplant intravenous fluid regimen; this varies by paediatric transplant centre and individual clinician. Many paediatric transplant centres use Hartmann's fluid or 0.9% sodium chloride intraoperatively, followed by a combination of 0.45% sodium chloride and 0.9% sodium chloride with varying concentrations of glucose postoperatively. Some centres use 4.5% Human Albumin Solution in addition(2). The variability in standard clinical practice has arisen from the lack of evidence to inform choice of intravenous fluid in this patient population. For this pragmatic trial, permitting variation in the control arm is necessary to achieve a robust comparison of the intervention to current clinical care.

Outcome measures

The primary outcome measure is acute hyponatraemia within the first 72 hours post-transplant, defined as laboratory plasma sodium concentration <135mmol/L confirmed on a concurrent blood gas sample where feasible.

Secondary outcome measures are as follows:

- Symptoms of acute hyponatraemia (nausea, vomiting, headache, seizures) within the first 72 hours post-transplant.
- The degree of fluid overload experienced (defined as maximal proportional acute increase in patient weight from pre-transplant weight in the first 72 hours post-transplant)
- Time to discharge from hospital (measured from transplant operation start time ("knife to skin") in days)
- Transplant kidney function at 1, 3, 7 and 90 days (eGFR calculated using creatinine-based univariate Schwartz formula(22))
- Other electrolyte abnormalities within the first 72 hours post transplantation:
    - hypernatraemia (defined as plasma sodium concentration >145mmol/l)
    - hyperkalaemia (defined as plasma potassium concentration >5.5mmol/L)
    - hypokalaemia (defined as plasma potassium concentration <3.5mmol/L)
    - non-anion-gap acidosis (defined as plasma bicarbonate <20mmol/L and anion gap < 20mmol/L)
    - hyperglycaemia (defined as random blood glucose >5.5 mmol/L)

For peer review only

- hypomagnesaemia (defined as plasma magnesium concentration <0.7 mmol/L)
- hyperchloraemia (defined as plasma chloride concentration >107mmol/L)
- excessive rate of reduction in plasma sodium concentration (defined as >1mmol/L/hour averaged over 6 hours)
- excessive magnitude of reduction in plasma sodium concentration (defined as >10mmol/L from pre-transplant level)

- Maximum and minimum systolic blood pressure (normalised to age and height percentile) sustained on 3 repeated values on each day, for 3 days post-transplant
- Number of changes in intravenous fluid composition within the first 72 hours post-transplant

Eligibility criteria

Inclusion Criteria

1. Patients under 18 years of age at the time of transplantation with valid patient/parental consent
2. Patients receiving a kidney-only transplant from either a living or deceased donor, in a participating UK centre

Exclusion Criteria

1. Multi-organ transplant recipients

Screening, recruitment and randomisation

All patients <18 years old awaiting kidney transplantation, from either a deceased or living donor, will be screened for participation. The trial will be discussed during the process of preparation for kidney transplant in order to allow adequate time for families to consider participation.

Age-appropriate consent/assent will be taken from children/young people and/or a parent/guardian as appropriate.

Participants will be randomised 1:1 to the intervention or control groups via an online system(23). Randomisation will be stratified by transplant centre and patient weight (<20kg vs ≥20kg); and will further be balanced within blocks of varying, undisclosed sizes. The randomisation list will be produced by the trial statistician using SAS statistical software.

Recruitment Process Evaluation (Qualitative Sub-Study)

A process evaluation will explore patients' and families' perceptions of the participant information, recruitment and consent process and acceptability of the trial.

At the time of initial approach for participation in PLUTO, families will be given a questionnaire (Figure 2a), completion of which will be voluntary. A follow-up questionnaire will be given to families who provide initial consent for the PLUTO trial at Day 4-7 post-transplant (Figure 2b).

For peer review only

Both questionnaires include an option to opt-in to being contacted for a telephone interview. Interviews will explore experience of recruitment and consent, participant information, motivation for participation (or reasons for not participating) and reasons for changes in decision making.

Treatment

Participants randomised to the intervention arm will receive either Plasma-Lyte® 148 or Plasma-Lyte® 148 & Glucose 5%, which can be administered interchangeably at the discretion of the clinical team, both intraoperatively and postoperatively (Figure 1). Participants randomised to the control arm will receive the standard intravenous fluids used at the site. Neither Plasma-Lyte® 148 nor Plasma-Lyte® 148 & Glucose 5% should be administered in the control arm. The volume and rate of intravenous fluid (control or intervention) will be determined at the discretion of the treating clinician. The decision to discontinue intravenous fluid and switch to enteral fluid will be made by the treating clinician.

Apart from intravenous fluid composition, all participants will receive routine clinical transplant care as summarised in the schedule of procedures (Table 2).

Data Collection

Baseline Assessments

Baseline assessments comprise those undertaken as part of routine clinical care prior to kidney transplant:

- **Eligibility Checklist**
- **Baseline Form (pre-transplant)**: details of the end stage kidney disease (cause, dialysis type, dialysis start date, native urine output), actual patient weight (kg), patient height (cm), blood pressure.

Baseline demographic data (patient age, sex, ethnicity) will be obtained directly from the UK Transplant Registry (UKTR), held by NHS Blood and Transplant (NHSBT).

On Day of Transplant

- **Randomisation Form (on day of transplant)**: randomisation date/time, randomisation ID number, treatment arm
- **Pre-transplant blood test results** (Table 3)

Post-Transplant (72 hours)

The 72 hour post-transplant period starts at the time of transplant operation “knife to skin”.

- **Transplant Operation Form**: operation start time, duration, graft placement
- **Fluids**: all fluid input / output, and all patient weight measurements in the first 72 hours post-transplant
- **Blood Products**: all blood products administered in the first 72 hours post-transplant

For peer review only

- **Medications**: all inotropes, diuretics, immunosuppressives, insulin, anti-emetics and electrolyte supplements administered in the first 72 hours post-transplant
- **Symptom Assessment**: daily symptom assessment for nausea, vomiting, headache, pain score, seizures. Pain is graded using an age-appropriate scale (FLACC or Wong-Baker)(24, 25).
- **Blood Pressure**: Maximum and minimum systolic blood pressure sustained on 3 repeated values on each day, for 3 days post-transplant
- **Blood Test Results**: all results to 72 hours post-transplant will be collected (Table 3).

Follow-Up Data Collection

- **Post-Transplant Form**: need for dialysis post-transplant, creatinine at Day 7 post-transplant (+/- 1 day)
- **Hospital Discharge**: date of discharge from hospital

No additional outpatient visits will be required for the research study. Plasma creatinine concentration at Month 3 (+/- 14 days) will be obtained directly from the UKTR held by NHSBT.

Statistical Plan

Sample size

The sample size required is 144 participants randomised. The baseline incidence was determined by analysis of 76 paediatric transplants performed at Great Ormond Street Hospital between 1 January 2015 and 31 March 2018 which showed that 45 children (59%) experienced hyponatraemia within the first 72 hours post-transplant when managed with 0.45% saline 5% glucose fluid(2). The effect size was estimated from the Cochrane systematic review comparing isotonic to hypotonic maintenance fluid in children, in which a 50% reduction in risk of hyponatraemia was shown(11). A greater effect size is anticipated because the rate of fluid delivery to children following kidney transplant significantly exceeds maintenance requirements(9). Using the 50% effect size from the Cochrane review, the assumed incidence of hyponatraemia would be reduced to 29.5%. A two-sided test with 90% power, 5% type I error and 1:1 allocation and allowing for two formal interim analyses for harm or benefit would require 128 participants. After allowing for 10% attrition, for example if a transplant cannot proceed due to a positive cross match, the total number of participants required is 144.

Interim analyses

A group sequential design with O'Brien-Fleming boundaries will inform early stopping of the trial in the case of strong evidence of harm or benefit, while preserving the overall 5% type I error rate. Two interim analyses to test for harm or benefit will be conducted after 70 and 100 participants have been recruited into the trial with primary outcome data. The stopping rules will be used as a guideline, alongside the other safety data available to the DMC, and used as part of their overall assessment of the trial.

For peer review only

Analysis

The analyses will be described in detail in a full Statistical Analysis Plan. This section summarises the main issues.

The population used for efficacy analyses will be a modified intention to treat analysis which will include all randomised participants who receive a transplant, as although unlikely it would be illogical to include any participant who did not receive a transplant after being randomised. This will be the primary analysis for the trial though the primary outcome will also be analysed per protocol.

Characteristics of all randomised patients will be tabulated by arm of the trial to describe the cohort. A CONSORT diagram will be presented to show the flow of patients through the trial.

Primary outcome: proportion of patients who experience hyponatraemia (defined as plasma sodium concentration <135mmol/L) within 72 hours post-transplant. This will be analysed using a logistic regression model adjusting for donor type (living vs deceased donor), patient weight (<20kg vs ≥20kg pre-transplant) and transplant centre as a random effect. Subgroup and sensitivity analyses will be used to assess whether the treatment effect differs according to the fluid regimen used in the standard arm.

Secondary outcomes:
The secondary outcomes which assess the incidence of other electrolyte and acid-base disturbance within 72 hours post transplantation (hyperkalaemia, hypokalaemia, hypernatraemia, acidosis, hypomagnesaemia, hyperglycaemia and excessive rate and magnitude of fall in plasma sodium concentration) will all be analysed using the same model as the primary outcome. Exact logistic regression will be used to compare instances of nausea/vomiting, headache and seizures. Adjusted normal linear regression models will be used to assess differences in the continuous secondary outcomes and an adjusted Cox proportional hazards model will be used to assess differences in time to discharge. The number of changes in intravenous fluid composition within the first 72 hours post-transplant will be summarised by treatment arm.

Any missing primary and secondary outcome data will be summarised. Primary and secondary outcome measures will not be imputed, and these will be treated as missing data and excluded from the relevant analyses. If outcome data are missing for more than 25% of participants, outcomes will not be reported. To explore if missing values have an undue impact on the primary outcome result, a sensitivity analysis using multiple imputation will be performed if the primary outcome is missing in more than 5% of the participants included in the modified intention to treat analysis.

Patient and Public Involvement Statement

A kidney transplant patient and her mother are key members of the study team, and five local families with children awaiting kidney transplant helped shape the research plan. The NIHR Great Ormond Street Hospital Young Person's Advisory Group for research (YPAG) co-designed patient information resources for this study. We have planned wider patient and public involvement at a national level together with the NHS Blood and Transplant Patient and Public Advisory Group.

Ethics, Safety and Dissemination

Ethical Compliance

For peer review only

The Investigator will ensure that this trial is conducted in accordance with the principles of the Declaration of Helsinki, ICH Good Clinical Practice Guidelines and in accordance with the terms and conditions of the ethical approval given to the trial. A favourable opinion has been given by a Research Ethics Committee (HRA reference 19/LO/1866).

Safety

Adverse Events and Expected Adverse Drug Reactions

Non-serious AEs will not be reported. Plasma-Lyte 148 is a licensed product and therefore adverse reactions which are expected (assessed against the Summary of Product Characteristics(18)) and not serious do not require recording.

SAEs will be notified within 24 hours and monitored regularly by the data monitoring committee (DMC) to ensure ongoing safety of trial participants.

The SAEs listed below are anticipated in this patient group, and have therefore been excluded from trial safety reporting:

1. **Surgical complications:**
   a. Return to theatre for exploration
   b. Haemorrhage
   c. Blood transfusion
   d. Transplant obstruction/dilatation/hydronephrosis
   e. Thrombosis (thrombosis of transplant, DVT, Pulmonary embolus, arteriovenous fistula)
   f. Transplant kidney arteriovenous fistula
   g. Transplant nephrectomy
   h. Haematuria
   i. cystoscopy for evacuation of haematoma or stent removal
   j. renal artery stenosis
   k. bowel injury, obstruction or ischaemia
   l. lymphocele/collection +/-drainage
   m. hernia
   n. urine leak
   o. nephrostomy

2. **Infective complications:**
   a. Viraemia (EBV, CMV, JC and BK viraemia)
   b. Bacteraemia
   c. Fever

3. **Immunological complications:**
   a. Acute rejection
   b. Transplant kidney biopsy

4. **Medication related complications:**
   a. Calcineurin inhibitor nephrotoxicity and neurotoxicity
   b. New onset diabetes

For peer review only

5. **Other:**
   a. Delayed graft function/primary non-function
   b. Thrombocytopenia/ thrombotic microangiopathy

Trial oversight

The Trial Management Group (TMG) will be responsible for the day to day running and management of the trial.

The Trial Steering Committee (TSC) will provide overall supervision for the trial and provide advice through its Independent Chair. The ultimate decision on continuation of the trial lies with the TSC.

The Data Monitoring Committee (DMC) will be responsible for safety monitoring. Recommendations made by the DMC to alter the conduct of the study will be forwarded to the TSC for a final decision. The Sponsor will forward such decisions to regulatory authorities, as appropriate. The DMC will be independent of the study team and will have no direct involvement in other aspects of the trial.

Dissemination

The final study data set will be analysed, and results published as soon as possible following completion of study follow-up, final data checks and database lock. The Trial Management Group will form the basis of the Writing Committee and will advise on the nature of publications.

Study findings will be presented to academic and non-academic groups. The patient and public involvement group will play an important part in disseminating the study findings into the public domain. Dissemination to non-academic audiences including service users, commissioners, clinicians and service providers will be facilitated through the use of existing networks e.g. email lists, social media.

All research teams and PPI members involved in the study will be invited to a close out meeting to discuss the findings of the study.

Open access, peer reviewed academic outputs and research reports together with associated summaries and key findings will be produced for funders, policy makers and NHS audiences and held on the study website.

Any publications arising from this study will adhere to the NIHR funding and support outputs guidance.

Figure Captions

Figure 1: PLUTO trial flowchart
Flowchart summarising study processes

Figure 2: Qualitative Sub-Study Questionnaires
Process evaluation questionnaires to explore patients' and families' perceptions of the participant information, recruitment and consent process and acceptability of the trial

For peer review only

References

1. Roach JP, Bock ME, Goebel J. Pediatric kidney transplantation. Seminars in pediatric surgery. 2017;26(4):233-40.
2. Hayes W, Longley C, Scanlon N, Bryant W, Stojanovic J, Kessaris N, et al. Plasma electrolyte imbalance in pediatric kidney transplant recipients. Pediatr Transplant. 2019:e13411.
3. Drake K, Nehus E, Goebel J. Hyponatremia, hypo-osmolality, and seizures in children early post-kidney transplant. Pediatr Transplant. 2015;19(7):698-703.
4. Cansick J, Rees L, Koffman G, Van't Hoff W, Bockenhauer D. A fatal case of cerebral oedema with hyponatraemia and massive polyuria after renal transplantation. Pediatr Nephrol. 2009;24(6):1231-4.
5. Arieff AI, Ayus JC, Fraser CL. Hyponatraemia and death or permanent brain damage in healthy children. Bmj. 1992;304(6836):1218-22.
6. Ayus JC, Achinger SG, Arieff A. Brain cell volume regulation in hyponatremia: role of sex, age, vasopressin, and hypoxia. Am J Physiol Renal Physiol. 2008;295(3):F619-24.
7. Gankam Kengne F, Decaux G. Hyponatremia and the Brain. Kidney Int Rep. 2018;3(1):24-35.
8. Giuliani C, Peri A. Effects of Hyponatremia on the Brain. J Clin Med. 2014;3(4):1163-77.
9. Coupe N, O'Brien M, Gibson P, de Lima J. Anesthesia for pediatric renal transplantation with and without epidural analgesia--a review of 7 years experience. Paediatr Anaesth. 2005;15(3):220-8.
10. Forsythe J. Report of the Inquiry into hyponatraemia related deaths. 2018.
11. McNab S, Ware RS, Neville KA, Choong K, Coulthard MG, Duke T, et al. Isotonic versus hypotonic solutions for maintenance intravenous fluid administration in children. Cochrane Database Syst Rev. 2014(12):CD009457.
12. Foster BA, Tom D, Hill V. Hypotonic versus isotonic fluids in hospitalized children: a systematic review and meta-analysis. J Pediatr. 2014;165(1):163-9 e2.
13. Wang J, Xu E, Xiao Y. Isotonic versus hypotonic maintenance IV fluids in hospitalized children: a meta-analysis. Pediatrics. 2014;133(1):105-13.
14. Choong K, Kho ME, Menon K, Bohn D. Hypotonic versus isotonic saline in hospitalised children: a systematic review. Arch Dis Child. 2006;91(10):828-35.
15. McNab S, Duke T, South M, Babl FE, Lee KJ, Arnup SJ, et al. 140 mmol/L of sodium versus 77 mmol/L of sodium in maintenance intravenous fluid therapy for children in hospital (PIMS): a randomised controlled double-blind trial. Lancet. 2015;385(9974):1190-7.
16. Weiss SL, Babl FE, Dalziel SR, Balamuth F. Is chloride worth its salt? Intensive care medicine. 2019;45(2):275-7.
17. National Institute for Health and Care Excellence. Intravenous fluid therapy in children and young people in hospital. 2016.
18. Electronic medicines compendium. Plasma-Lyte 148 (pH 7.4) solution for infusion.
19. Wan S, Roberts MA, Mount P. Normal saline versus lower-chloride solutions for kidney transplantation. Cochrane Database Syst Rev. 2016(8):CD010741.
20. Weinberg L, Harris L, Bellomo R, Ierino FL, Story D, Eastwood G, et al. Effects of intraoperative and early postoperative normal saline or Plasma-Lyte 148(R) on hyperkalaemia in deceased donor renal transplantation: a double-blind randomized trial. British journal of anaesthesia. 2017;119(4):606-15.
21. Adwaney A, Randall DW, Blunden MJ, Prowle JR, Kirwan CJ. Perioperative Plasma-Lyte use reduces the incidence of renal replacement therapy and hyperkalaemia following renal transplantation when compared with 0.9% saline: a retrospective cohort study. Clin Kidney J. 2017;10(6):838-44.
22. Schwartz GJ, Munoz A, Schneider MF, Mak RH, Kaskel F, Warady BA, et al. New equations to estimate GFR in children with CKD. Journal of the American Society of Nephrology : JASN. 2009;20(3):629-37.

For peer review only

23. Lee ST, Cho H. Fluid overload and outcomes in neonates receiving continuous renal replacement therapy. Pediatr Nephrol. 2016;31(11):2145-52.
24. Merkel SI, Voepel-Lewis T, Shayevitz JR, Malviya S. The FLACC: a behavioral scale for scoring postoperative pain in young children. Pediatr Nurs. 1997;23(3):293-7.
25. Garra G, Singer AJ, Taira BR, Chohan J, Cardoz H, Chisena E, et al. Validation of the Wong-Baker FACES Pain Rating Scale in pediatric emergency department patients. Acad Emerg Med. 2010;17(1):50-4.

Acknowledgements
This study is sponsored by Great Ormond Street Hospital for Children NHS Foundation Trust. The study was supported by the Digital Research Environment Team at Great Ormond Street Hospital.

Our thanks to Maya and Sylwia Olejnik-Antkowiak, patient and parent representatives who have contributed to the design of this study, and to the NIHR Great Ormond Street Hospital Young Person's Advisory Group for research (YPAG) for co-designing patient information resources.

Authors' contributions
WNH developed the idea for this study, drafted this manuscript and has oversight of the study with mentorship from MJP. WNH, EL, LP, HT and MJP applied for funding and developed the trial protocol. JW developed the recruitment process evaluation. NK, SO-A, MA, WB, AS and SDM are all study co-investigators and contributed to the writing of this protocol. EL, CF and LP are the trial manager, clinical operations manager and trial statistician, respectively, at the NHSBT CTU and contributed to the writing of the protocol and study planning and approval process. All authors read and approved the final manuscript.

Funding statement
This study is funded by the National Institute for Health Research Research for Patient Benefit programme (project reference number NIHR200512). The views expressed are those of the author(s) and not necessarily those of the NIHR or the Department of Health and Social Care.

Competing interests statement
None declared

For peer review only

Table 1: Participating Paediatric Kidney Transplant Centres

Trust	Principal Investigator
Royal Belfast Hospital for Sick Children	Dr Mairead Convery
Birmingham Childrens Hospital	Dr Mordi Mourah
Bristol Royal Hospital for Children	Dr Jan Dudley
Great Ormond Street Hospital	Dr Wesley Hayes
Evelina London Children's Hospital	Dr Nick Ware
Leeds Children's Hospital	Dr Pallavi Yadav
Royal Manchester Children's Hospital	Dr Mohan Shenoy
Great North Children's Hospital	Dr Michal Malina
Nottingham Children's Hospital	Dr Martin Christian
University Hospital of Wales*	Dr Shivaram Hegde
University Hospital Southampton*	Dr Shuman Haq
Alder Hey Children's Hospital*	Dr Henry Morgan

* Participant identification centre

review only

Table 2: Schedule of Procedures

No additional tests are required for PLUTO trial participants. This table summarises the data required at each timepoint:

	Screening	Baseline (Pre-Tx)	At Tx	Day 1 Post-Tx	Day 2 Post-Tx	Day 3 Post-Tx	Day 7 Post-Tx	Month 3 Post-Tx	Hospital Discharge
**ENROLMENT**									
Qualitative Sub-Study – give Q1	X								
Eligibility assessment	X								
Informed consent		X							
Baseline Characteristics		X							
Re-confirm consent/assent			X						
Randomisation			X						
Qualitative Sub-Study – give Q2							X		
**TREATMENT**									
Administration of trial fluid			X	X	X	X			
**ASSESSMENTS**									
Transplant Operation Data			X						
Blood Results**		X	All results for 72 hours from transplant 'knife-to-skin'						
Blood Gas Results**		X	All results for 72 hours from transplant 'knife-to-skin'						
Patient Weight		X	All weights for 72 hours from transplant 'knife-to-skin'						
Fluid Data			All fluid for 72 hours from transplant 'knife-to-skin'						
Medication Data			All meds* for 72 hours from transplant 'knife-to-skin'						
Symptom Assessment				X	X	X			
Dialysis				X	X	X			
Blood Pressure		X		X	X	X			
Safety Reporting			X	X	X	X			
Transplant Graft							X	X***	

For peer review only

Function									
Discharge Date									X

*Data must be collected on all inotropes, diuretics, immunosuppressives, insulin, anti-emetics and electrolyte supplements administered during this 72 hour period.

**Blood Results and Venus Blood Gas data will be obtained directly from the Trust Pathology System (*where possible*), anonymised and then transferred to the Digital Research Environment at GOSH.

***Data will be obtained directly from the UKTR, held by NHSBT(e.g. patient ethnicity, blood group, donor details, matching details, transplant graft function).

For peer review only

Table 3. Blood parameters

Test	Preferred Units
Plasma sodium level	mmol/L
Plasma potassium level	mmol/L
Plasma urea level	mmol/L
Plasma creatinine level	micromol/L
Plasma chloride level	mmol/L
Plasma bicarbonate level	mmol/L
Plasma magnesium level	mmol/L
Plasma calcium level	mmol/L
Plasma inorganic phosphate level	mmol/L
Blood glucose level	mmol/L
Haemoglobin mass concentration in blood	g/L
Blood gas pH	-
Blood gas – bicarbonate	mmol/L
Blood gas – chloride	mmol/L
Blood gas – sodium	mmol/L
Blood gas – potassium	mmol/L
Blood gas - ionised calcium	mmol/L
Blood gas – glucose	mmol/l

Figure 1 – PLUTO trial flowchart

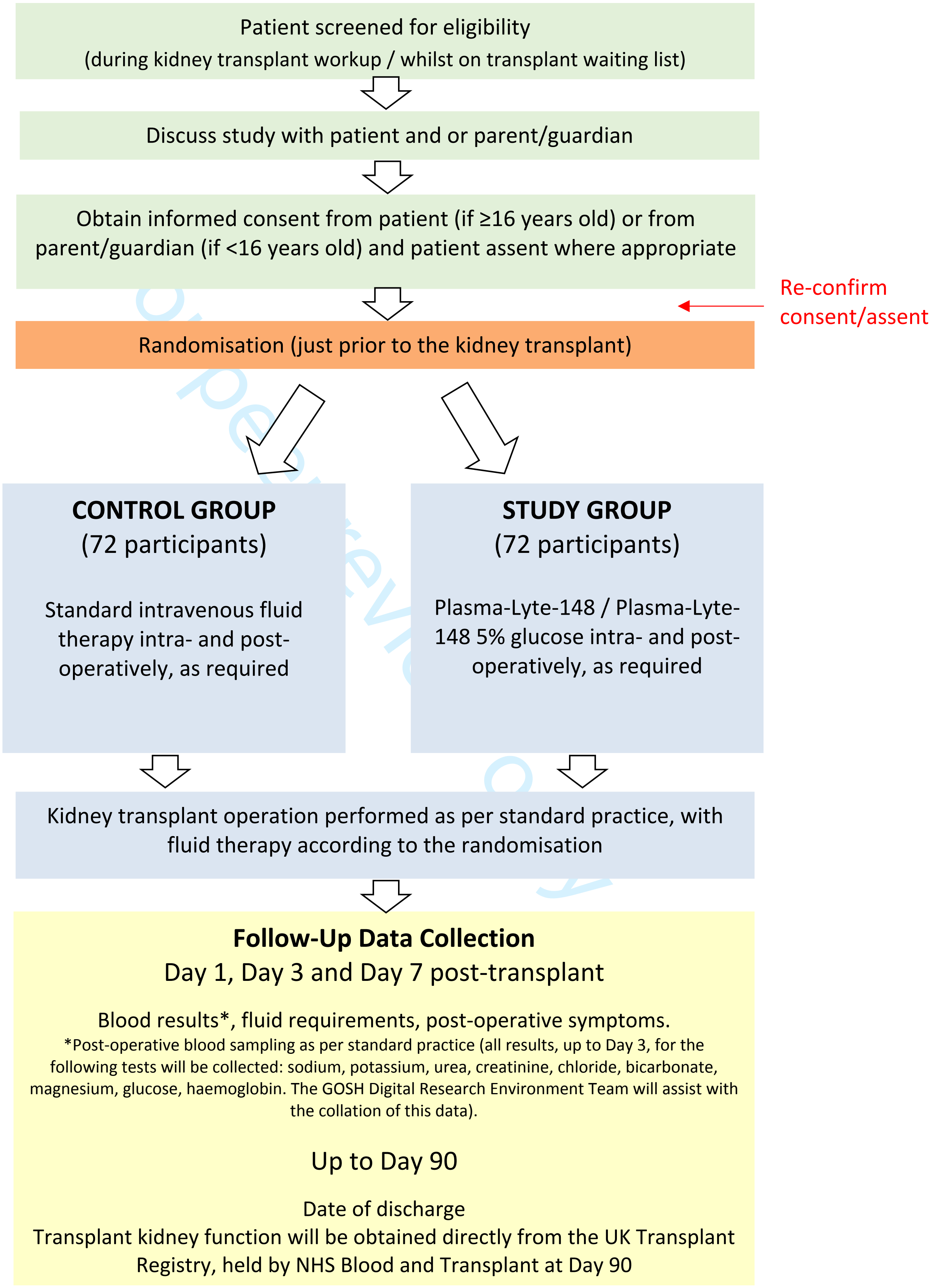

For peer review only - http://bmjopen.bmj.com/site/about/guidelines.xhtml

Figure 2: Qualitative Sub-Study Questionnaires

QUESTIONNAIRE 1 – PRE-TRANSPLANT

	Strongly agree	Agree	Neither agree nor disagree	Disagree	Strongly disagree
I was satisfied with how I was **approached** about PLUTO	○	○	○	○	○
I understood the **information** that I received from the doctor/research nurse about PLUTO	○	○	○	○	○
I had sufficient opportunity to ask **questions** about PLUTO	○	○	○	○	○
Any questions I had about PLUTO were answered in a way that I could **understand**	○	○	○	○	○
I was satisfied with the **consent** process for PLUTO	○	○	○	○	○
I had enough **time** to think about whether or not to consent to my child participating in PLUTO	○	○	○	○	○

QUESTIONNAIRE 2 – POST-TRANSPLANT

	Strongly agree	Agree	Neither agree nor disagree	Disagree	Strongly disagree
I was satisfied with how I was **approached** about PLUTO on the night of transplant	○	○	○	○	○
I remembered the **information** I had previously been given about PLUTO	○	○	○	○	○
I had sufficient opportunity to ask **questions** about PLUTO on the night of transplant	○	○	○	○	○
Any questions I had about PLUTO were answered in a way that I could **understand**	○	○	○	○	○
I was satisfied with the **re-consent** process for PLUTO on the night of transplant	○	○	○	○	○
I was happy with my original **decision** to consent to my child participating in PLUTO	○	○	○	○	○

BMJ Open

Protocol for multi-centre randomised controlled trial: Plasma-Lyte Usage and assessment of kidney Transplant Outcomes in children (PLUTO)

Journal:	*BMJ Open*
Manuscript ID	bmjopen-2021-055595.R1
Article Type:	Protocol
Date Submitted by the Author:	16-Dec-2021
Complete List of Authors:	Hayes, Wesley; Great Ormond Street Hospital for Children NHS Foundation Trust; University College London<br>Laing, Emma; NHS Blood and Transplant, Clinical Trials Unit<br>Foley, Claire; NHSBT Clinical Trials Unit<br>Pankhurst, Laura; NHS Blood and Transplant<br>Thomas, Helen; NHSBT Clinical Trials Unit<br>Hume-Smith, Helen; Great Ormond Street Hospital for Children NHS Foundation Trust; University College London<br>Marks, Dr. Stephen; Great Ormond Street Hospital For Children NHS Trust, Paediatric Nephrology<br>Kessaris, Nicos; Guy's Hospital, Department of Nephrology and Transplantation<br>Bryant, William A.; Great Ormond Street Hospital for Children NHS Foundation Trust<br>Spiridou, Anastassia; Great Ormond Street Hospital for Children NHS Foundation Trust<br>Wray, Jo; Great Ormond Street Hospital for Children, Heart and Lung Directorate<br>Peters, Mark; Great Ormond Street Hospital For Children NHS Trust, Paediatric Intensive Care Unit; University College London, Institute of Child Health
**Primary Subject Heading**:	Renal medicine
Secondary Subject Heading:	Paediatrics
Keywords:	Renal transplantation < NEPHROLOGY, Paediatric nephrology < NEPHROLOGY, TRANSPLANT MEDICINE

SCHOLARONE™
Manuscripts

Protocol for multi-centre randomised controlled trial: Plasma-Lyte Usage and assessment of kidney Transplant Outcomes in children (PLUTO)

Wesley N Hayes[1,2], Emma Laing[3], Claire Foley[3], Laura Pankhurst[3], Helen Thomas[3], Helen Hume-Smith[1,2], Stephen D Marks[1,2], Nicos Kessaris[1,4], William Bryant[1], Anastassia Spiridou[1], Jo Wray[1], Mark J Peters[2,1]

1. Great Ormond Street Hospital for Children NHS Foundation Trust, and NIHR Biomedical Research Centre, WC1N 3JH, London, United Kingdom
2. University College London, Great Ormond Street Institute for Child Health, London, UK
3. National Health Service Blood and Transplant Clinical Trials Unit, UK
4. Guys and St Thomas NHS Foundation Trust, London UK

Corresponding Author:

Wesley N Hayes, Wesley.Hayes@gosh.nhs.uk

ORCID-ID:

Wesley Hayes (ORCID 0000-0002-8091-6945)

Stephen D Marks (ORCID 0000-0001-9850-8352)

Emma Laing (ORCID 0000-0002-8309-0990)

Anastassia Spiridou (ORCID 0000-0001-6576-0244)

Mark J Peters (ORCID 0000-0003-3653-4808)

For peer review only

Abstract

Introduction

Acute electrolyte and acid-base imbalance is experienced by many children following kidney transplantation. When severe this can lead to complications including seizures, cerebral oedema and death. Relatively large volumes of intravenous fluid are administered to children perioperatively in order to establish perfusion to the donor kidney, the majority of which are from living and deceased adult donors. Hypotonic intravenous fluid is commonly used in the post-transplant period due to clinicians' concerns about the sodium, chloride and potassium content of isotonic alternatives when administered in large volumes.

Plasma-Lyte-148 is an isotonic, balanced intravenous fluid that contains sodium, chloride, potassium and magnesium with concentrations equivalent to those of plasma. There is a physiological basis to expect that Plasma-Lyte-148 will reduce the incidence of clinically significant electrolyte and acid-base abnormalities in children following kidney transplantation compared to current practice.

The aim of the PLUTO trial is to determine whether the incidence of clinically significantly abnormal plasma electrolyte levels in paediatric kidney transplant recipients will be different with the use of Plasma-Lyte-148 compared to intravenous fluid currently administered.

Methods and analysis

PLUTO is a pragmatic, open-label, randomised controlled trial comparing Plasma-Lyte 148 to current care in paediatric kidney transplant recipients, conducted in 9 UK paediatric kidney transplant centres.

A total of 144 children receiving kidney transplants will be randomised to receive either PlasmaLyte-148 (the intervention) intra- and postoperatively, or current fluid. Apart from intravenous fluid composition, all participants will receive standard clinical transplant care.

The primary outcome measure is acute hyponatraemia in the first 72 hours post-transplant, defined as laboratory plasma sodium concentration <135mmol/L. Secondary outcomes include symptoms of acute hyponatraemia, other electrolyte and acid base imbalances and transplant kidney function.

The primary outcome will be analysed using a logistic regression model adjusting for donor type (living vs deceased donor), patient weight (<20kg vs ≥20kg pre-transplant) and transplant centre as a random effect.

Ethics and dissemination

The trial received Health Research Authority approval on 20 January 2020. Findings will be presented to academic groups via national and international conferences and peer-reviewed journals. The patient and public involvement group will play an important part in disseminating the study findings into the public domain.

Registration details

EudraCT Number: 2019-003025-22

ISRCTN: 16586164

For peer review only

Strengths and Limitations of this study

- This is the first randomised controlled trial to evaluate balanced isotonic intravenous fluid in paediatric kidney transplant recipients
- A process evaluation will be used to tailor the trial to optimise children and families' experience of participating
- Variation in current clinical practice between centres and clinicians makes standardisation of fluid administration in the control arm impractical, which could potentially affect the effect size.
- Blinding was not considered necessary due to the objective nature of outcome measures

For peer review only

Introduction

Kidney transplantation is the treatment of choice for children with end stage kidney disease(1). Currently, acute electrolyte and acid-base imbalance is experienced by many paediatric recipients in the post-operative period(2). Abnormalities include acute hyponatraemia, hyperkalaemia and metabolic acidosis; smaller recipients of deceased donor kidneys are most at risk(2).

Acute hyponatraemia is closely associated with clinical complications in children after transplant: cerebral oedema, seizures(3) and death(4). Up to 8% of children suffer hyponatraemia-related seizures following kidney transplant(3). Pre-pubertal children are at particular risk of complications from acute hyponatraemia because of the relatively low ratio of cerebrospinal fluid and brain volume compared to older children and adults(5, 6). Acute hyponatraemia also predicts symptoms such as nausea and vomiting, headaches, breathlessness and reduced conscious level(7, 8).

Several factors predispose children to acute hyponatraemia following kidney transplant:

Firstly, relatively large volumes of intravenous fluid are administered perioperatively in order to establish and maintain perfusion to the adult donor kidney(9). Secondly, hypotonic intravenous fluid is predominantly administered postoperatively in paediatric kidney transplant centres(2). Thirdly, the post-operative period is characterised by an acute antidiuretic response which limits water excretion(8, 10).

Children receiving isotonic fluid as opposed to hypotonic fluid for indications other than kidney transplant experience less hyponatraemia(11-15). A randomised trial in children with sepsis comparing balanced fluid to 0.9% sodium chloride is currently underway (Pragmatic Pediatric Trial of Balanced vs Normal Saline Fluid in Sepsis (PRoMPT BOLUS) study, Clinicaltrials.gov NCT03340805)(16). National guidance now recommends isotonic fluid for children in the general paediatric setting(17). However, this guidance has not been adopted in paediatric transplant care because of a view that the period following kidney transplantation differs from other situations in terms of the volume of fluid administered and the associated sodium and chloride load. Hence, hypotonic fluid is still routinely administered to children following kidney transplantation(2, 3) and the use of isotonic fluid in this context requires evaluation.

Plasma-Lyte-148 is an isotonic, balanced intravenous fluid(18). It is licenced in children and contains sodium, chloride, potassium, magnesium with concentrations equivalent to those of plasma. There is a physiological basis to expect that Plasma-Lyte-148 will reduce the incidence of clinically significant electrolyte and acid-base abnormalities in children following kidney transplantation compared to current standard fluid.

A perceived risk of hyperkalaemia from the potassium content of Plasma-Lyte concerns some paediatric nephrologists, although a systematic review in adult kidney transplant recipients demonstrated less hyperkalaemia and acidosis with Plasma-Lyte compared to 0.9% sodium chloride(19). A randomised trial(20) and a retrospective analysis in adult kidney transplant recipients(21) found a lower risk of hyperkalaemia with Plasma-Lyte-148 compared to 0.9% sodium chloride.

Currently, there is equipoise regarding use of balanced isotonic fluid vs hypotonic and chloride rich solutions for children undergoing kidney transplant.

For peer review only

The aim of the PLUTO trial is to determine whether the incidence of abnormal and potentially dangerous plasma electrolyte levels in paediatric kidney transplant recipients will be different with the use of Plasma-Lyte-148 compared to current standard intravenous fluids.

Methods and Analysis

Study design and setting

PLUTO is an open-label randomised controlled trial comparing Plasma-Lyte 148 (the intervention) to current intravenous fluids in paediatric kidney transplant recipients, conducted in 9 UK paediatric kidney transplant centres plus 3 participant identification centres (Table 1). The study started on 8 June 2020, with planned completion 31 Oct 2022.

Intervention and comparator

Plasma-Lyte® 148 solution for infusion and Plasma-Lyte® 148 & Glucose 5% w/v solution for infusion are licenced in children and used in paediatric intensive care, surgical, and general paediatric wards. They contain sodium 140mmol/l, chloride 98mmol/l, potassium 5mmol/l, magnesium 1.5mmol/l and gluconate/acetate buffer to maintain physiological pH(18). Both solutions can be administered interchangeably within the intervention arm.

The comparator is the current paediatric kidney transplant intravenous fluid regimen; this varies by paediatric transplant centre and individual clinician. Many paediatric transplant centres use Hartmann's fluid or 0.9% sodium chloride intraoperatively, followed by a combination of 0.45% sodium chloride and 0.9% sodium chloride with varying concentrations of glucose postoperatively. Some centres use 4.5% Human Albumin Solution in addition(2). The variability in standard clinical practice has arisen from the lack of evidence to inform choice of intravenous fluid in this patient population. For this pragmatic trial, permitting variation in the control arm is necessary to achieve a robust comparison of the intervention to current clinical care.

Outcome measures

The primary outcome measure is acute hyponatraemia within the first 72 hours post-transplant, defined as laboratory plasma sodium concentration <135mmol/L confirmed on a concurrent blood gas sample where feasible.

Secondary outcome measures are as follows:

- Symptoms of acute hyponatraemia (nausea, vomiting, headache, seizures) within the first 72 hours post-transplant.
- The degree of fluid overload experienced (defined as maximal proportional acute increase in patient weight from pre-transplant weight in the first 72 hours post-transplant)
- Time to discharge from hospital (measured from transplant operation start time ("knife to skin") in days)
- Transplant kidney function at 1, 3, 7 and 90 days (eGFR calculated using creatinine-based univariate Schwartz formula(22))
- Other electrolyte abnormalities within the first 72 hours post transplantation:
  - hypernatraemia (defined as plasma sodium concentration >145mmol/l)
  - hyperkalaemia (defined as plasma potassium concentration >5.5mmol/L)

For peer review only

- hypokalaemia (defined as plasma potassium concentration <3.5mmol/L)
- non-anion-gap acidosis (defined as plasma bicarbonate <20mmol/L and anion gap < 20mmol/L)
- hyperglycaemia (defined as random blood glucose >5.5 mmol/L)
- hypomagnesaemia (defined as plasma magnesium concentration <0.7 mmol/L)
- hyperchloraemia (defined as plasma chloride concentration >107mmol/L)
- excessive rate of reduction in plasma sodium concentration (defined as >1mmol/L/hour averaged over 6 hours)
- excessive magnitude of reduction in plasma sodium concentration (defined as >10mmol/L from pre-transplant level)

- Maximum and minimum systolic blood pressure (normalised to age and height percentile) sustained on 3 repeated values on each day, for 3 days post-transplant
- Number of changes in intravenous fluid composition within the first 72 hours post-transplant

Eligibility criteria

Inclusion Criteria

1. Patients under 18 years of age at the time of transplantation with valid patient/parental consent
2. Patients receiving a kidney-only transplant from either a living or deceased donor, in a participating UK centre

Exclusion Criteria

1. Multi-organ transplant recipients

Screening, recruitment and randomisation

All patients <18 years old awaiting kidney transplantation, from either a deceased or living donor, will be screened for participation. The trial will be discussed during the process of preparation for kidney transplant in order to allow adequate time for families to consider participation. The potential benefits and risks of study participation are included in the study information sheets.

In accordance with Clinical Trial Regulations, patients aged under 16 years are deemed to be ‘minors’ and an appropriate adult must give consent on behalf of the child. The wishes of the child must form part of the decision-making process and the child’s assent will be sought. For young people aged 16 – 18 years, their informed consent will be taken.

Participants will be randomised 1:1 to the intervention or control groups via an online system(23). Randomisation will be stratified by transplant centre and patient weight (<20kg vs ≥20kg); and will further be balanced within blocks of varying, undisclosed sizes. The randomisation list will be produced by the trial statistician using SAS statistical software.

Treatment

Participants randomised to the intervention arm will receive either Plasma-Lyte® 148 or Plasma-Lyte® 148 & Glucose 5%, which can be administered interchangeably at the discretion of the clinical team, both intraoperatively and postoperatively (Figure 1). Participants randomised to the control

For peer review only

arm will receive the standard intravenous fluids used at the site. Neither Plasma-Lyte® 148 nor Plasma-Lyte® 148 & Glucose 5% should be administered in the control arm. The volume and rate of intravenous fluid (control or intervention) will be determined at the discretion of the treating clinician. The decision to discontinue intravenous fluid and switch to enteral fluid will be made by the treating clinician.

Apart from intravenous fluid composition, all participants will receive routine clinical transplant care as summarised in the schedule of procedures (Table 2).

Recruitment Process Evaluation (Qualitative Sub-Study)

A process evaluation will explore patients' and families' perceptions of the participant information, recruitment and consent process and acceptability of the trial.

At the time of initial approach for participation in PLUTO, families will be given a questionnaire (Figure 2a), completion of which will be voluntary. A follow-up questionnaire will be given to families who provide initial consent for the PLUTO trial at Day 4-7 post-transplant (Figure 2b).

Both questionnaires include an option to opt-in to being contacted for a telephone interview. Interviews will explore experience of recruitment and consent, participant information, motivation for participation (or reasons for not participating) and reasons for changes in decision making.

Data Collection

Baseline Assessments

Baseline assessments comprise those undertaken as part of routine clinical care prior to kidney transplant:

- **Eligibility Checklist**
- **Baseline Form (pre-transplant)**: details of the end stage kidney disease (cause, dialysis type, dialysis start date, native urine output), actual patient weight (kg), patient height (cm), blood pressure.

Baseline demographic data (patient age, sex, ethnicity) will be obtained directly from the UK Transplant Registry (UKTR), held by NHS Blood and Transplant (NHSBT).

On Day of Transplant

- **Randomisation Form (on day of transplant)**: randomisation date/time, randomisation ID number, treatment arm
- **Pre-transplant blood test results** (Table 3)

Post-Transplant (72 hours)

The 72 hour post-transplant period starts at the time of transplant operation "knife to skin".

- **Transplant Operation Form**: operation start time, duration, graft placement

For peer review only

- **Fluids**: all fluid input / output, and all patient weight measurements in the first 72 hours post-transplant
- **Blood Products**: all blood products administered in the first 72 hours post-transplant
- **Medications**: all inotropes, diuretics, immunosuppressives, insulin, anti-emetics and electrolyte supplements administered in the first 72 hours post-transplant
- **Symptom Assessment**: daily symptom assessment for nausea, vomiting, headache, pain score, seizures. Pain is graded using an age-appropriate scale (FLACC or Wong-Baker)(24, 25).
- **Blood Pressure**: Maximum and minimum systolic blood pressure sustained on 3 repeated values on each day, for 3 days post-transplant
- **Blood Test Results**: all results to 72 hours post-transplant will be collected (Table 3).

Follow-Up Data Collection

- **Post-Transplant Form**: need for dialysis post-transplant, creatinine at Day 7 post-transplant (+/- 1 day)
- **Hospital Discharge**: date of discharge from hospital

No additional outpatient visits will be required for the research study. Plasma creatinine concentration at Month 3 (+/- 14 days) will be obtained directly from the UKTR held by NHSBT.

Statistical Plan

Sample size

The sample size required is 144 participants randomised. The baseline incidence was determined by analysis of 76 paediatric transplants performed at Great Ormond Street Hospital between 1 January 2015 and 31 March 2018 which showed that 45 children (59%) experienced hyponatraemia within the first 72 hours post-transplant when managed with 0.45% saline 5% glucose fluid(2). The effect size was estimated from the Cochrane systematic review comparing isotonic to hypotonic maintenance fluid in children, in which a 50% reduction in risk of hyponatraemia was shown(11). A greater effect size is anticipated because the rate of fluid delivery to children following kidney transplant significantly exceeds maintenance requirements(9). Using the 50% effect size from the Cochrane review, the assumed incidence of hyponatraemia would be reduced to 29.5%. A two-sided test with 90% power, 5% type I error and 1:1 allocation and allowing for two formal interim analyses for harm or benefit would require 128 participants. After allowing for 10% attrition, for example if a transplant cannot proceed due to a positive cross match, the total number of participants required is 144.

Interim analyses

A group sequential design with O'Brien-Fleming boundaries will inform early stopping of the trial in the case of strong evidence of harm or benefit, while preserving the overall 5% type I error rate. Two interim analyses to test for harm or benefit will be conducted after 70 and 100 participants have been recruited into the trial with primary outcome data. The stopping rules will be used as a

For peer review only

guideline, alongside the other safety data available to the DMC, and used as part of their overall assessment of the trial.

Analysis

The analyses will be described in detail in a full Statistical Analysis Plan. This section summarises the main issues.

The population used for efficacy analyses will be a modified intention to treat analysis which will include all randomised participants who receive a transplant, as although unlikely it would be illogical to include any participant who did not receive a transplant after being randomised. This will be the primary analysis for the trial though the primary outcome will also be analysed per protocol.

Characteristics of all randomised patients will be tabulated by arm of the trial to describe the cohort. A CONSORT diagram will be presented to show the flow of patients through the trial.

Primary outcome: proportion of patients who experience hyponatraemia (defined as plasma sodium concentration <135mmol/L) within 72 hours post-transplant. This will be analysed using a logistic regression model adjusting for donor type (living vs deceased donor), patient weight (<20kg vs ≥20kg pre-transplant) and transplant centre as a random effect. Subgroup and sensitivity analyses will be used to assess whether the treatment effect differs according to the fluid regimen used in the standard arm.

Secondary outcomes:
The secondary outcomes which assess the incidence of other electrolyte and acid-base disturbance within 72 hours post transplantation (hyperkalaemia, hypokalaemia, hypernatraemia, acidosis, hypomagnesaemia, hyperglycaemia and excessive rate and magnitude of fall in plasma sodium concentration) will all be analysed using the same model as the primary outcome. Exact logistic regression will be used to compare instances of nausea/vomiting, headache and seizures. Adjusted normal linear regression models will be used to assess differences in the continuous secondary outcomes and an adjusted Cox proportional hazards model will be used to assess differences in time to discharge. The number of changes in intravenous fluid composition within the first 72 hours post-transplant will be summarised by treatment arm.

Any missing primary and secondary outcome data will be summarised. Primary and secondary outcome measures will not be imputed, and these will be treated as missing data and excluded from the relevant analyses. If outcome data are missing for more than 25% of participants, outcomes will not be reported. To explore if missing values have an undue impact on the primary outcome result, a sensitivity analysis using multiple imputation will be performed if the primary outcome is missing in more than 5% of the participants included in the modified intention to treat analysis.

Patient and Public Involvement
A kidney transplant patient and her mother are co-applicants for the grant and members of the Trial Steering Committee (TSC). They make sure that the trial remains patient-centered throughout its delivery and will lead the dissemination of results to patients and families. The research plan was also shaped by a workshop with the Young Persons' Advisory Group at Great Ormond Street Hospital, and involvement of five families with children awaiting kidney transplantation who advised on the burden of intervention.

For peer review only

Ethics, Safety and Dissemination

Ethical Compliance

The Investigator will ensure that this trial is conducted in accordance with the principles of the Declaration of Helsinki, ICH Good Clinical Practice Guidelines and in accordance with the terms and conditions of the ethical approval given to the trial. A favourable opinion has been given by a Research Ethics Committee (HRA reference 19/LO/1866).

Safety

Adverse Events and Expected Adverse Drug Reactions

Non-serious AEs will not be reported. Plasma-Lyte 148 is a licensed product and therefore adverse reactions which are expected (assessed against the Summary of Product Characteristics(18)) and not serious do not require recording.

SAEs will be notified within 24 hours and monitored regularly by the data monitoring committee (DMC) to ensure ongoing safety of trial participants.

The SAEs listed below are anticipated in this patient group, and have therefore been excluded from trial safety reporting:

1. **Surgical complications:**
    a. Return to theatre for exploration
    b. Haemorrhage
    c. Blood transfusion
    d. Transplant obstruction/dilatation/hydronephrosis
    e. Thrombosis (thrombosis of transplant, DVT, Pulmonary embolus, arteriovenous fistula)
    f. Transplant kidney arteriovenous fistula
    g. Transplant nephrectomy
    h. Haematuria
    i. cystoscopy for evacuation of haematoma or stent removal
    j. renal artery stenosis
    k. bowel injury, obstruction or ischaemia
    l. lymphocele/collection +/-drainage
    m. hernia
    n. urine leak
    o. nephrostomy

2. **Infective complications:**
    a. Viraemia (EBV, CMV, JC and BK viraemia)
    b. Bacteraemia
    c. Fever

3. **Immunological complications:**
    a. Acute rejection
    b. Transplant kidney biopsy

For peer review only

4. **Medication related complications:**
    a. Calcineurin inhibitor nephrotoxicity and neurotoxicity
    b. New onset diabetes

5. **Other:**
    a. Delayed graft function/primary non-function
    b. Thrombocytopenia/ thrombotic microangiopathy

Trial oversight

The Trial Management Group (TMG) will be responsible for the day to day running and management of the trial.

The Trial Steering Committee (TSC) will provide overall supervision for the trial and provide advice through its Independent Chair. The ultimate decision on continuation of the trial lies with the TSC.

The Data Monitoring Committee (DMC) will be responsible for safety monitoring. Recommendations made by the DMC to alter the conduct of the study will be forwarded to the TSC for a final decision. The Sponsor will forward such decisions to regulatory authorities, as appropriate. The DMC will be independent of the study team and will have no direct involvement in other aspects of the trial.

Dissemination

The final study data set will be analysed, and results published as soon as possible following completion of study follow-up, final data checks and database lock. The Trial Management Group will form the basis of the Writing Committee and will advise on the nature of publications.

Study findings will be presented to academic and non-academic groups. The patient and public involvement group will play an important part in disseminating the study findings into the public domain. Dissemination to non-academic audiences including service users, commissioners, clinicians and service providers will be facilitated through the use of existing networks e.g. email lists, social media.

All research teams and PPI members involved in the study will be invited to a close out meeting to discuss the findings of the study.

Open access, peer reviewed academic outputs and research reports together with associated summaries and key findings will be produced for funders, policy makers and NHS audiences and held on the study website.

Any publications arising from this study will adhere to the NIHR funding and support outputs guidance.

References

1. Roach JP, Bock ME, Goebel J. Pediatric kidney transplantation. Seminars in pediatric surgery. 2017;26(4):233-40.

For peer review only

2. Hayes W, Longley C, Scanlon N, Bryant W, Stojanovic J, Kessaris N, et al. Plasma electrolyte imbalance in pediatric kidney transplant recipients. Pediatr Transplant. 2019:e13411.
3. Drake K, Nehus E, Goebel J. Hyponatremia, hypo-osmolality, and seizures in children early post-kidney transplant. Pediatr Transplant. 2015;19(7):698-703.
4. Cansick J, Rees L, Koffman G, Van't Hoff W, Bockenhauer D. A fatal case of cerebral oedema with hyponatraemia and massive polyuria after renal transplantation. Pediatr Nephrol. 2009;24(6):1231-4.
5. Arieff AI, Ayus JC, Fraser CL. Hyponatraemia and death or permanent brain damage in healthy children. Bmj. 1992;304(6836):1218-22.
6. Ayus JC, Achinger SG, Arieff A. Brain cell volume regulation in hyponatremia: role of sex, age, vasopressin, and hypoxia. Am J Physiol Renal Physiol. 2008;295(3):F619-24.
7. Gankam Kengne F, Decaux G. Hyponatremia and the Brain. Kidney Int Rep. 2018;3(1):24-35.
8. Giuliani C, Peri A. Effects of Hyponatremia on the Brain. J Clin Med. 2014;3(4):1163-77.
9. Coupe N, O'Brien M, Gibson P, de Lima J. Anesthesia for pediatric renal transplantation with and without epidural analgesia--a review of 7 years experience. Paediatr Anaesth. 2005;15(3):220-8.
10. Forsythe J. Report of the Inquiry into hyponatraemia related deaths. 2018.
11. McNab S, Ware RS, Neville KA, Choong K, Coulthard MG, Duke T, et al. Isotonic versus hypotonic solutions for maintenance intravenous fluid administration in children. Cochrane Database Syst Rev. 2014(12):CD009457.
12. Foster BA, Tom D, Hill V. Hypotonic versus isotonic fluids in hospitalized children: a systematic review and meta-analysis. J Pediatr. 2014;165(1):163-9 e2.
13. Wang J, Xu E, Xiao Y. Isotonic versus hypotonic maintenance IV fluids in hospitalized children: a meta-analysis. Pediatrics. 2014;133(1):105-13.
14. Choong K, Kho ME, Menon K, Bohn D. Hypotonic versus isotonic saline in hospitalised children: a systematic review. Arch Dis Child. 2006;91(10):828-35.
15. McNab S, Duke T, South M, Babl FE, Lee KJ, Arnup SJ, et al. 140 mmol/L of sodium versus 77 mmol/L of sodium in maintenance intravenous fluid therapy for children in hospital (PIMS): a randomised controlled double-blind trial. Lancet. 2015;385(9974):1190-7.
16. Weiss SL, Babl FE, Dalziel SR, Balamuth F. Is chloride worth its salt? Intensive care medicine. 2019;45(2):275-7.
17. National Institute for Health and Care Excellence. Intravenous fluid therapy in children and young people in hospital. 2016.
18. Electronic medicines compendium. Plasma-Lyte 148 (pH 7.4) solution for infusion.
19. Wan S, Roberts MA, Mount P. Normal saline versus lower-chloride solutions for kidney transplantation. Cochrane Database Syst Rev. 2016(8):CD010741.
20. Weinberg L, Harris L, Bellomo R, Ierino FL, Story D, Eastwood G, et al. Effects of intraoperative and early postoperative normal saline or Plasma-Lyte 148(R) on hyperkalaemia in deceased donor renal transplantation: a double-blind randomized trial. British journal of anaesthesia. 2017;119(4):606-15.
21. Adwaney A, Randall DW, Blunden MJ, Prowle JR, Kirwan CJ. Perioperative Plasma-Lyte use reduces the incidence of renal replacement therapy and hyperkalaemia following renal transplantation when compared with 0.9% saline: a retrospective cohort study. Clin Kidney J. 2017;10(6):838-44.
22. Schwartz GJ, Munoz A, Schneider MF, Mak RH, Kaskel F, Warady BA, et al. New equations to estimate GFR in children with CKD. Journal of the American Society of Nephrology : JASN. 2009;20(3):629-37.
23. Lee ST, Cho H. Fluid overload and outcomes in neonates receiving continuous renal replacement therapy. Pediatr Nephrol. 2016;31(11):2145-52.
24. Merkel SI, Voepel-Lewis T, Shayevitz JR, Malviya S. The FLACC: a behavioral scale for scoring postoperative pain in young children. Pediatr Nurs. 1997;23(3):293-7.

For peer review only

25. Garra G, Singer AJ, Taira BR, Chohan J, Cardoz H, Chisena E, et al. Validation of the Wong-Baker FACES Pain Rating Scale in pediatric emergency department patients. Acad Emerg Med. 2010;17(1):50-4.

Acknowledgements

This study is sponsored by Great Ormond Street Hospital for Children NHS Foundation Trust. The study was supported by the Digital Research Environment Team at Great Ormond Street Hospital.

Our thanks to Maya and Sylwia Olejnik-Antkowiak, patient and parent representatives who have contributed to the design of this study, and to the NIHR Great Ormond Street Hospital Young Person's Advisory Group for research (YPAG) for co-designing patient information resources.

Authors' contributions

WNH developed the idea for this study, drafted this manuscript and has oversight of the study with mentorship from MJP. WNH, EL, LP, HT and MJP applied for funding and developed the trial protocol. JW developed the recruitment process evaluation. NK, SO-A, MA, WB, AS and SDM are all study co-investigators and contributed to the writing of this protocol. EL, CF and LP are the trial manager, clinical operations manager and trial statistician, respectively, at the NHSBT CTU and contributed to the writing of the protocol and study planning and approval process. All authors read and approved the final manuscript.

Funding statement

This study is funded by the National Institute for Health Research Research for Patient Benefit programme (project reference number NIHR200512). The views expressed are those of the author(s) and not necessarily those of the NIHR or the Department of Health and Social Care.

Competing interests statement

None declared

For peer review only

Table 1: Participating Paediatric Kidney Transplant Centres

Trust	Principal Investigator
Royal Belfast Hospital for Sick Children	Dr Mairead Convery
Birmingham Childrens Hospital	Dr Mordi Mourah
Bristol Royal Hospital for Children	Dr Jan Dudley
Great Ormond Street Hospital	Dr Wesley Hayes
Evelina London Children's Hospital	Dr Nick Ware
Leeds Children's Hospital	Dr Pallavi Yadav
Royal Manchester Children's Hospital	Dr Mohan Shenoy
Great North Children's Hospital	Dr Michal Malina
Nottingham Children's Hospital	Dr Martin Christian
University Hospital of Wales*	Dr Shivaram Hegde
University Hospital Southampton*	Dr Shuman Haq
Alder Hey Children's Hospital*	Dr Henry Morgan

* Participant identification centre

review only

Table 2: Schedule of Procedures

No additional tests are required for PLUTO trial participants. This table summarises the data required at each timepoint:

	Screening	Baseline (Pre-Tx)	At Tx	Day 1 Post-Tx	Day 2 Post-Tx	Day 3 Post-Tx	Day 7 Post-Tx	Month 3 Post-Tx	Hospital Discharge
**ENROLMENT**									
Qualitative Sub-Study – give Q1	X								
Eligibility assessment	X								
Informed consent		X							
Baseline Characteristics		X							
Re-confirm consent/assent			X						
Randomisation			X						
Qualitative Sub-Study – give Q2							X		
**TREATMENT**									
Administration of trial fluid			X	X	X	X			
**ASSESSMENTS**									
Transplant Operation Data			X						
Blood Results**		X	All results for 72 hours from transplant 'knife-to-skin'						
Blood Gas Results**		X	All results for 72 hours from transplant 'knife-to-skin'						
Patient Weight		X	All weights for 72 hours from transplant 'knife-to-skin'						
Fluid Data			All fluid for 72 hours from transplant 'knife-to-skin'						
Medication Data			All meds* for 72 hours from transplant 'knife-to-skin'						
Symptom Assessment				X	X	X			
Dialysis				X	X	X			
Blood Pressure		X		X	X	X			
Safety Reporting			X	X	X	X			
Transplant Graft							X	X***	

For peer review only

Function									
Discharge Date									X

*Data must be collected on all inotropes, diuretics, immunosuppressives, insulin, anti-emetics and electrolyte supplements administered during this 72 hour period.
**Blood Results and Venus Blood Gas data will be obtained directly from the Trust Pathology System (*where possible*), anonymised and then transferred to the Digital Research Environment at GOSH.
***Data will be obtained directly from the UKTR, held by NHSBT(e.g. patient ethnicity, blood group, donor details, matching details, transplant graft function).

For peer review only

Table 3. Blood parameters

**Test**	**Preferred Units**
Plasma sodium level	mmol/L
Plasma potassium level	mmol/L
Plasma urea level	mmol/L
Plasma creatinine level	micromol/L
Plasma chloride level	mmol/L
Plasma bicarbonate level	mmol/L
Plasma magnesium level	mmol/L
Plasma calcium level	mmol/L
Plasma inorganic phosphate level	mmol/L
Blood glucose level	mmol/L
Haemoglobin mass concentration in blood	g/L
Blood gas pH	-
Blood gas – bicarbonate	mmol/L
Blood gas – chloride	mmol/L
Blood gas – sodium	mmol/L
Blood gas – potassium	mmol/L
Blood gas - ionised calcium	mmol/L
Blood gas – glucose	mmol/l

Figure 1 – PLUTO trial flowchart

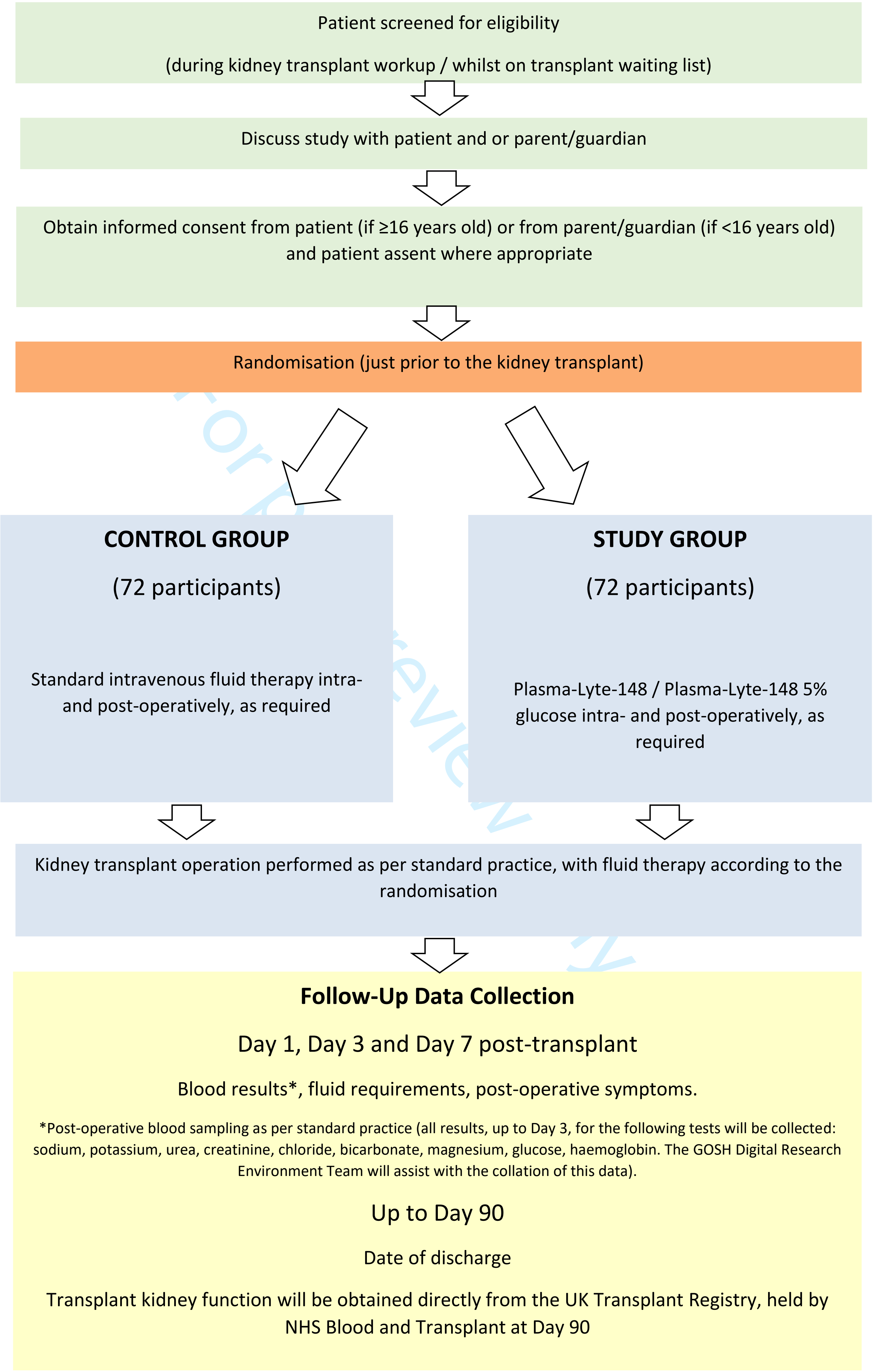

Figure 2: Qualitative Sub-Study Questionnaires

QUESTIONNAIRE 1 – PRE-TRANSPLANT

	Strongly agree	Agree	Neither agree nor disagree	Disagree	Strongly disagree
I was satisfied with how I was **approached** about PLUTO	◯	◯	◯	◯	◯
I understood the **information** that I received from the doctor/research nurse about PLUTO	◯	◯	◯	◯	◯
I had sufficient opportunity to ask **questions** about PLUTO	◯	◯	◯	◯	◯
Any questions I had about PLUTO were answered in a way that I could **understand**	◯	◯	◯	◯	◯
I was satisfied with the **consent** process for PLUTO	◯	◯	◯	◯	◯
I had enough **time** to think about whether or not to consent to my child participating in PLUTO	◯	◯	◯	◯	◯

For peer review only

QUESTIONNAIRE 2 – POST-TRANSPLANT

	Strongly agree	Agree	Neither agree nor disagree	Disagree	Strongly disagree
I was satisfied with how I was **approached** about PLUTO on the night of transplant	◯	◯	◯	◯	◯
I remembered the **information** I had previously been given about PLUTO	◯	◯	◯	◯	◯
I had sufficient opportunity to ask **questions** about PLUTO on the night of transplant	◯	◯	◯	◯	◯
Any questions I had about PLUTO were answered in a way that I could **understand**	◯	◯	◯	◯	◯
I was satisfied with the **re-consent** process for PLUTO on the night of transplant	◯	◯	◯	◯	◯
I was happy with my original **decision** to consent to my child participating in PLUTO	◯	◯	◯	◯	◯

For peer review only

STANDARD PROTOCOL ITEMS: RECOMMENDATIONS FOR INTERVENTIONAL TRIALS

SPIRIT 2013 Checklist: Recommended items to address in a clinical trial protocol and related documents*

Section/item	Item No	Description	Addressed on page number
**Administrative information**			
Title	1	Descriptive title identifying the study design, population, interventions, and, if applicable, trial acronym	_____1________
Trial registration	2a	Trial identifier and registry name. If not yet registered, name of intended registry	_____ 2_______
	2b	All items from the World Health Organization Trial Registration Data Set	______2_______
Protocol version	3	Date and version identifier	Manuscript based on full trial protocol v2.0 dated 23 March 2021
Funding	4	Sources and types of financial, material, and other support	______13_______
Roles and responsibilities	5a	Names, affiliations, and roles of protocol contributors	________1_____
	5b	Name and contact information for the trial sponsor	_______13______
	5c	Role of study sponsor and funders, if any, in study design; collection, management, analysis, and interpretation of data; writing of the report; and the decision to submit the report for publication, including whether they will have ultimate authority over any of these activities	_______13_____

For peer review only

	5d	Composition, roles, and responsibilities of the coordinating centre, steering committee, endpoint adjudication committee, data management team, and other individuals or groups overseeing the trial, if applicable (see Item 21a for data monitoring committee)	______11_______

Introduction

Background and rationale	6a	Description of research question and justification for undertaking the trial, including summary of relevant studies (published and unpublished) examining benefits and harms for each intervention	____4&5_____
	6b	Explanation for choice of comparators	_____5_________
Objectives	7	Specific objectives or hypotheses	______5 (aim)__
Trial design	8	Description of trial design including type of trial (eg, parallel group, crossover, factorial, single group), allocation ratio, and framework (eg, superiority, equivalence, noninferiority, exploratory)	________5______

Methods: Participants, interventions, and outcomes

Study setting	9	Description of study settings (eg, community clinic, academic hospital) and list of countries where data will be collected. Reference to where list of study sites can be obtained	________5_____
Eligibility criteria	10	Inclusion and exclusion criteria for participants. If applicable, eligibility criteria for study centres and individuals who will perform the interventions (eg, surgeons, psychotherapists)	______6_______
Interventions	11a	Interventions for each group with sufficient detail to allow replication, including how and when they will be administered	______7_____
	11b	Criteria for discontinuing or modifying allocated interventions for a given trial participant (eg, drug dose change in response to harms, participant request, or improving/worsening disease)	_____7_____
	11c	Strategies to improve adherence to intervention protocols, and any procedures for monitoring adherence (eg, drug tablet return, laboratory tests)	N/A, intervention is delivered by clinical staff

	11d	Relevant concomitant care and interventions that are permitted or prohibited during the trial	_____7_____
Outcomes	12	Primary, secondary, and other outcomes, including the specific measurement variable (eg, systolic blood pressure), analysis metric (eg, change from baseline, final value, time to event), method of aggregation (eg, median, proportion), and time point for each outcome. Explanation of the clinical relevance of chosen efficacy and harm outcomes is strongly recommended	_____5&6_____
Participant timeline	13	Time schedule of enrolment, interventions (including any run-ins and washouts), assessments, and visits for participants. A schematic diagram is highly recommended (see Figure)	_____15________
Sample size	14	Estimated number of participants needed to achieve study objectives and how it was determined, including clinical and statistical assumptions supporting any sample size calculations	______8_______
Recruitment	15	Strategies for achieving adequate participant enrolment to reach target sample size	_______7______
**Methods: Assignment of interventions (for controlled trials)**			
Allocation:			
Sequence generation	16a	Method of generating the allocation sequence (eg, computer-generated random numbers), and list of any factors for stratification. To reduce predictability of a random sequence, details of any planned restriction (eg, blocking) should be provided in a separate document that is unavailable to those who enrol participants or assign interventions	_______6______
Allocation concealment mechanism	16b	Mechanism of implementing the allocation sequence (eg, central telephone; sequentially numbered, opaque, sealed envelopes), describing any steps to conceal the sequence until interventions are assigned	_______6______
Implementation	16c	Who will generate the allocation sequence, who will enrol participants, and who will assign participants to interventions	_______6______
Blinding (masking)	17a	Who will be blinded after assignment to interventions (eg, trial participants, care providers, outcome assessors, data analysts), and how	N/A – open label trial
	17b	If blinded, circumstances under which unblinding is permissible, and procedure for revealing a participant's allocated intervention during the trial	____N/A_____

Methods: Data collection, management, and analysis

Data collection methods	18a	Plans for assessment and collection of outcome, baseline, and other trial data, including any related processes to promote data quality (eg, duplicate measurements, training of assessors) and a description of study instruments (eg, questionnaires, laboratory tests) along with their reliability and validity, if known. Reference to where data collection forms can be found, if not in the protocol	______7_______
	18b	Plans to promote participant retention and complete follow-up, including list of any outcome data to be collected for participants who discontinue or deviate from intervention protocols	N/A, data collection only during hospitalisation, follow up data will be obtained from a patient registry
Data management	19	Plans for data entry, coding, security, and storage, including any related processes to promote data quality (eg, double data entry; range checks for data values). Reference to where details of data management procedures can be found, if not in the protocol	More details are included in the study's data management and statistical analysis plan - limited by word count in the manuscript
Statistical methods	20a	Statistical methods for analysing primary and secondary outcomes. Reference to where other details of the statistical analysis plan can be found, if not in the protocol	______9_______
	20b	Methods for any additional analyses (eg, subgroup and adjusted analyses)	______9_______
	20c	Definition of analysis population relating to protocol non-adherence (eg, as randomised analysis), and any statistical methods to handle missing data (eg, multiple imputation)	______9_______

Methods: Monitoring

Data monitoring	21a	Composition of data monitoring committee (DMC); summary of its role and reporting structure; statement of whether it is independent from the sponsor and competing interests; and reference to where further details about its charter can be found, if not in the protocol. Alternatively, an explanation of why a DMC is not needed	______11_______
	21b	Description of any interim analyses and stopping guidelines, including who will have access to these interim results and make the final decision to terminate the trial	_____9______
Harms	22	Plans for collecting, assessing, reporting, and managing solicited and spontaneously reported adverse events and other unintended effects of trial interventions or trial conduct	____10&11___
Auditing	23	Frequency and procedures for auditing trial conduct, if any, and whether the process will be independent from investigators and the sponsor	_____N/A____
**Ethics and dissemination**			
Research ethics approval	24	Plans for seeking research ethics committee/institutional review board (REC/IRB) approval	______2_______
Protocol amendments	25	Plans for communicating important protocol modifications (eg, changes to eligibility criteria, outcomes, analyses) to relevant parties (eg, investigators, REC/IRBs, trial participants, trial registries, journals, regulators)	______11______
Consent or assent	26a	Who will obtain informed consent or assent from potential trial participants or authorised surrogates, and how (see Item 32)	______6_______
	26b	Additional consent provisions for collection and use of participant data and biological specimens in ancillary studies, if applicable	___N/A______
Confidentiality	27	How personal information about potential and enrolled participants will be collected, shared, and maintained in order to protect confidentiality before, during, and after the trial	Available in the protocol and patient documents, - limited by word count in the manuscript

peer review only

For peer review only - http://bmjopen.bmj.com/site/about/guidelines.xhtml

Declaration of interests	28	Financial and other competing interests for principal investigators for the overall trial and each study site	______13_______
Access to data	29	Statement of who will have access to the final trial dataset, and disclosure of contractual agreements that limit such access for investigators	Details available on the ISRCTN registry – limited by word count in the manuscript
Ancillary and post-trial care	30	Provisions, if any, for ancillary and post-trial care, and for compensation to those who suffer harm from trial participation	Details included in the full trial protocol - limited by word count in the manuscript
Dissemination policy	31a	Plans for investigators and sponsor to communicate trial results to participants, healthcare professionals, the public, and other relevant groups (eg, via publication, reporting in results databases, or other data sharing arrangements), including any publication restrictions	______2_____
	31b	Authorship eligibility guidelines and any intended use of professional writers	_____N/A______
	31c	Plans, if any, for granting public access to the full protocol, participant-level dataset, and statistical code	_____N/A______
**Appendices**			
Informed consent materials	32	Model consent form and other related documentation given to participants and authorised surrogates	__Attached ___
Biological specimens	33	Plans for collection, laboratory evaluation, and storage of biological specimens for genetic or molecular analysis in the current trial and for future use in ancillary studies, if applicable	___N/A______

*It is strongly recommended that this checklist be read in conjunction with the SPIRIT 2013 Explanation & Elaboration for important clarification on the items. Amendments to the protocol should be tracked and dated. The SPIRIT checklist is copyrighted by the SPIRIT Group under the Creative Commons "Attribution-NonCommercial-NoDerivs 3.0 Unported" license.

Protocol for multi-centre randomised controlled trial: Plasma-Lyte Usage and assessment of kidney Transplant Outcomes in children (PLUTO)

Journal:	*BMJ Open*
Manuscript ID	bmjopen-2021-055595.R2
Article Type:	Protocol
Date Submitted by the Author:	21-Feb-2022
Complete List of Authors:	Hayes, Wesley; Great Ormond Street Hospital for Children NHS Foundation Trust; University College London<br>Laing, Emma; NHS Blood and Transplant, Clinical Trials Unit<br>Foley, Claire; NHSBT Clinical Trials Unit<br>Pankhurst, Laura; NHS Blood and Transplant<br>Thomas, Helen; NHSBT Clinical Trials Unit<br>Hume-Smith, Helen; Great Ormond Street Hospital for Children NHS Foundation Trust; University College London<br>Marks, Dr. Stephen; Great Ormond Street Hospital For Children NHS Trust, Paediatric Nephrology<br>Kessaris, Nicos; Guy's Hospital, Department of Nephrology and Transplantation<br>Bryant, William A.; Great Ormond Street Hospital for Children NHS Foundation Trust<br>Spiridou, Anastassia; Great Ormond Street Hospital for Children NHS Foundation Trust<br>Wray, Jo; Great Ormond Street Hospital for Children, Heart and Lung Directorate<br>Peters, Mark; Great Ormond Street Hospital For Children NHS Trust, Paediatric Intensive Care Unit; University College London, Institute of Child Health
**Primary Subject Heading**:	Renal medicine
Secondary Subject Heading:	Paediatrics
Keywords:	Renal transplantation < NEPHROLOGY, Paediatric nephrology < NEPHROLOGY, TRANSPLANT MEDICINE

SCHOLARONE™ Manuscripts

Protocol for multi-centre randomised controlled trial: Plasma-Lyte Usage and assessment of kidney Transplant Outcomes in children (PLUTO)

Wesley N Hayes[1,2], Emma Laing[3], Claire Foley[3], Laura Pankhurst[3], Helen Thomas[3], Helen Hume-Smith[1,2], Stephen D Marks[1,2], Nicos Kessaris[1,4], William Bryant[1], Anastassia Spiridou[1], Jo Wray[1], Mark J Peters[2,1]

1. Great Ormond Street Hospital for Children NHS Foundation Trust, and NIHR Biomedical Research Centre, WC1N 3JH, London, United Kingdom
2. University College London, Great Ormond Street Institute for Child Health, London, UK
3. National Health Service Blood and Transplant Clinical Trials Unit, UK
4. Guys and St Thomas NHS Foundation Trust, London UK

Corresponding Author:

Wesley N Hayes, Wesley.Hayes@gosh.nhs.uk

ORCID-ID:

Wesley Hayes (ORCID 0000-0002-8091-6945)

Stephen D Marks (ORCID 0000-0001-9850-8352)

Emma Laing (ORCID 0000-0002-8309-0990)

Anastassia Spiridou (ORCID 0000-0001-6576-0244)

Mark J Peters (ORCID 0000-0003-3653-4808)

For peer review only

Abstract

Introduction

Acute electrolyte and acid-base imbalance is experienced by many children following kidney transplantation. When severe this can lead to complications including seizures, cerebral oedema and death. Relatively large volumes of intravenous fluid are administered to children perioperatively in order to establish perfusion to the donor kidney, the majority of which are from living and deceased adult donors. Hypotonic intravenous fluid is commonly used in the post-transplant period due to clinicians' concerns about the sodium, chloride and potassium content of isotonic alternatives when administered in large volumes.

Plasma-Lyte-148 is an isotonic, balanced intravenous fluid that contains sodium, chloride, potassium and magnesium with concentrations equivalent to those of plasma. There is a physiological basis to expect that Plasma-Lyte-148 will reduce the incidence of clinically significant electrolyte and acid-base abnormalities in children following kidney transplantation compared to current practice.

The aim of the PLUTO trial is to determine whether the incidence of clinically significantly abnormal plasma electrolyte levels in paediatric kidney transplant recipients will be different with the use of Plasma-Lyte-148 compared to intravenous fluid currently administered.

Methods and analysis

PLUTO is a pragmatic, open-label, randomised controlled trial comparing Plasma-Lyte 148 to current care in paediatric kidney transplant recipients, conducted in 9 UK paediatric kidney transplant centres.

A total of 144 children receiving kidney transplants will be randomised to receive either PlasmaLyte-148 (the intervention) intra- and postoperatively, or current fluid. Apart from intravenous fluid composition, all participants will receive standard clinical transplant care.

The primary outcome measure is acute hyponatraemia in the first 72 hours post-transplant, defined as laboratory plasma sodium concentration <135mmol/L. Secondary outcomes include symptoms of acute hyponatraemia, other electrolyte and acid base imbalances and transplant kidney function.

The primary outcome will be analysed using a logistic regression model adjusting for donor type (living vs deceased donor), patient weight (<20kg vs ≥20kg pre-transplant) and transplant centre as a random effect.

Ethics and dissemination

The trial received Health Research Authority approval on 20 January 2020. Findings will be presented to academic groups via national and international conferences and peer-reviewed journals. The patient and public involvement group will play an important part in disseminating the study findings into the public domain.

Registration details

EudraCT Number: 2019-003025-22

ISRCTN: 16586164

For peer review only

Strengths and Limitations of this study

- This is the first randomised controlled trial to evaluate balanced isotonic intravenous fluid in paediatric kidney transplant recipients
- A process evaluation will be used to tailor the trial to optimise children and families' experience of participating
- Variation in current clinical practice between centres and clinicians makes standardisation of fluid administration in the control arm impractical, which could potentially affect the effect size.
- Blinding was not considered necessary due to the objective nature of outcome measures

For peer review only

Introduction

Kidney transplantation is the treatment of choice for children with end stage kidney disease(1). Currently, acute electrolyte and acid-base imbalance is experienced by many paediatric recipients in the post-operative period(2). Abnormalities include acute hyponatraemia, hyperkalaemia and metabolic acidosis; smaller recipients of deceased donor kidneys are most at risk(2).

Acute hyponatraemia is closely associated with clinical complications in children after transplant: cerebral oedema, seizures(3) and death(4). Up to 8% of children suffer hyponatraemia-related seizures following kidney transplant(3). Pre-pubertal children are at particular risk of complications from acute hyponatraemia because of the relatively low ratio of cerebrospinal fluid and brain volume compared to older children and adults(5, 6). Acute hyponatraemia also predicts symptoms such as nausea and vomiting, headaches, breathlessness and reduced conscious level(7, 8).

Several factors predispose children to acute hyponatraemia following kidney transplant:

Firstly, relatively large volumes of intravenous fluid are administered perioperatively in order to establish and maintain perfusion to the adult donor kidney(9). Secondly, hypotonic intravenous fluid is predominantly administered postoperatively in paediatric kidney transplant centres(2). Thirdly, the post-operative period is characterised by an acute antidiuretic response which limits water excretion(8, 10).

Children receiving isotonic fluid as opposed to hypotonic fluid for indications other than kidney transplant experience less hyponatraemia(11-15). A randomised trial in children with sepsis comparing balanced fluid to 0.9% sodium chloride is currently underway (Pragmatic Pediatric Trial of Balanced vs Normal Saline Fluid in Sepsis (PRoMPT BOLUS) study, Clinicaltrials.gov NCT03340805)(16). National guidance now recommends isotonic fluid for children in the general paediatric setting(17). However, this guidance has not been adopted in paediatric transplant care because of a view that the period following kidney transplantation differs from other situations in terms of the volume of fluid administered and the associated sodium and chloride load. Hence, hypotonic fluid is still routinely administered to children following kidney transplantation(2, 3) and the use of isotonic fluid in this context requires evaluation.

Plasma-Lyte-148 is an isotonic, balanced intravenous fluid(18). It is licenced in children and contains sodium, chloride, potassium, magnesium with concentrations equivalent to those of plasma. There is a physiological basis to expect that Plasma-Lyte-148 will reduce the incidence of clinically significant electrolyte and acid-base abnormalities in children following kidney transplantation compared to current standard fluid.

A perceived risk of hyperkalaemia from the potassium content of Plasma-Lyte concerns some paediatric nephrologists, although a systematic review in adult kidney transplant recipients demonstrated less hyperkalaemia and acidosis with Plasma-Lyte compared to 0.9% sodium chloride(19). A randomised trial(20) and a retrospective analysis in adult kidney transplant recipients(21) found a lower risk of hyperkalaemia with Plasma-Lyte-148 compared to 0.9% sodium chloride.

Currently, there is equipoise regarding use of balanced isotonic fluid vs hypotonic and chloride rich solutions for children undergoing kidney transplant.

The aim of the PLUTO trial is to determine whether the incidence of abnormal and potentially dangerous plasma electrolyte levels in paediatric kidney transplant recipients will be different with the use of Plasma-Lyte-148 compared to current standard intravenous fluids.

For peer review only

Methods and Analysis

Study design and setting

PLUTO is an open-label randomised controlled trial comparing Plasma-Lyte 148 (the intervention) to current intravenous fluids in paediatric kidney transplant recipients, conducted in 9 UK paediatric kidney transplant centres plus 3 participant identification centres (Table 1). The study started on 8 June 2020, with planned completion 31 Oct 2022.

Intervention and comparator

Plasma-Lyte® 148 solution for infusion and Plasma-Lyte® 148 & Glucose 5% w/v solution for infusion are licenced in children and used in paediatric intensive care, surgical, and general paediatric wards. They contain sodium 140mmol/l, chloride 98mmol/l, potassium 5mmol/l, magnesium 1.5mmol/l and gluconate/acetate buffer to maintain physiological pH(Table 2)(18). Both solutions can be administered interchangeably within the intervention arm.

The comparator is the current paediatric kidney transplant intravenous fluid regimen; this varies by paediatric transplant centre and individual clinician. Many paediatric transplant centres use Hartmann's fluid or 0.9% sodium chloride intraoperatively, followed by a combination of 0.45% sodium chloride and 0.9% sodium chloride with varying concentrations of glucose postoperatively. Some centres use 4.5% Human Albumin Solution in addition(2). The variability in standard clinical practice has arisen from the lack of evidence to inform choice of intravenous fluid in this patient population. For this pragmatic trial, permitting variation in the control arm is necessary to achieve a robust comparison of the intervention to current clinical care.

Outcome measures

The primary outcome measure is acute hyponatraemia within the first 72 hours post-transplant, defined as laboratory plasma sodium concentration <135mmol/L confirmed on a concurrent blood gas sample where feasible.

Secondary outcome measures are as follows:

- Symptoms of acute hyponatraemia (nausea, vomiting, headache, seizures) within the first 72 hours post-transplant.
- The degree of fluid overload experienced (defined as maximal proportional acute increase in patient weight from pre-transplant weight in the first 72 hours post-transplant)
- Time to discharge from hospital (measured from transplant operation start time ("knife to skin") in days)
- Transplant kidney function at 1, 3, 7 and 90 days (eGFR calculated using creatinine-based univariate Schwartz formula(22))
- Other electrolyte abnormalities within the first 72 hours post transplantation:
    - hypernatraemia (defined as plasma sodium concentration >145mmol/l)
    - hyperkalaemia (defined as plasma potassium concentration >5.5mmol/L)
    - hypokalaemia (defined as plasma potassium concentration <3.5mmol/L)
    - non-anion-gap acidosis (defined as plasma bicarbonate <20mmol/L and anion gap < 20mmol/L)

For peer review only

  - hyperglycaemia (defined as random blood glucose >5.5 mmol/L)
  - hypomagnesaemia (defined as plasma magnesium concentration <0.7 mmol/L)
  - hyperchloraemia (defined as plasma chloride concentration >107mmol/L)
  - excessive rate of reduction in plasma sodium concentration (defined as >1mmol/L/hour averaged over 6 hours)
  - excessive magnitude of reduction in plasma sodium concentration (defined as >10mmol/L from pre-transplant level)
- Maximum and minimum systolic blood pressure (normalised to age and height percentile) sustained on 3 repeated values on each day, for 3 days post-transplant
- Number of changes in intravenous fluid composition within the first 72 hours post-transplant

Eligibility criteria

Inclusion Criteria

1. Patients under 18 years of age at the time of transplantation with valid patient/parental consent
2. Patients receiving a kidney-only transplant from either a living or deceased donor, in a participating UK centre

Exclusion Criteria

1. Multi-organ transplant recipients

Screening, recruitment and randomisation

All patients <18 years old awaiting kidney transplantation, from either a deceased or living donor, will be screened for participation. The trial will be discussed during the process of preparation for kidney transplant in order to allow adequate time for families to consider participation. The potential benefits and risks of study participation are included in the study information sheets.

In accordance with Clinical Trial Regulations, patients aged under 16 years are deemed to be 'minors' and an appropriate adult must give consent on behalf of the child. The wishes of the child must form part of the decision-making process and the child's assent will be sought. For young people aged 16 – 18 years, their informed consent will be taken.

Participants will be randomised 1:1 to the intervention or control groups via an online system(23). Randomisation will be stratified by transplant centre and patient weight (<20kg vs ≥20kg); and will further be balanced within blocks of varying, undisclosed sizes. The randomisation list will be produced by the trial statistician using SAS statistical software.

Treatment

Participants randomised to the intervention arm will receive either Plasma-Lyte® 148 or Plasma-Lyte® 148 & Glucose 5%, which can be administered interchangeably at the discretion of the clinical team, both intraoperatively and postoperatively (Figure 1). Participants randomised to the control arm will receive the standard intravenous fluids used at the site. Neither Plasma-Lyte® 148 nor Plasma-Lyte® 148 & Glucose 5% should be administered in the control arm. The volume and rate of intravenous fluid (control or intervention) will be determined at the discretion of the treating clinician. The

For peer review only

decision to discontinue intravenous fluid and switch to enteral fluid will be made by the treating clinician.

Apart from intravenous fluid composition, all participants will receive routine clinical transplant care as summarised in the schedule of procedures (Table 3).

Recruitment Process Evaluation (Qualitative Sub-Study)

A process evaluation will explore patients' and families' perceptions of the participant information, recruitment and consent process and acceptability of the trial.

At the time of initial approach for participation in PLUTO, families will be given a questionnaire (Figure 2a), completion of which will be voluntary. A follow-up questionnaire will be given to families who provide initial consent for the PLUTO trial at Day 4-7 post-transplant (Figure 2b).

Both questionnaires include an option to opt-in to being contacted for a telephone interview. Interviews will explore experience of recruitment and consent, participant information, motivation for participation (or reasons for not participating) and reasons for changes in decision making.

Data Collection

Baseline Assessments

Baseline assessments comprise those undertaken as part of routine clinical care prior to kidney transplant:

- **Eligibility Checklist**
- **Baseline Form (pre-transplant)**: details of the end stage kidney disease (cause, dialysis type, dialysis start date, native urine output), actual patient weight (kg), patient height (cm), blood pressure.

Baseline demographic data (patient age, sex, ethnicity) will be obtained directly from the UK Transplant Registry (UKTR), held by NHS Blood and Transplant (NHSBT).

On Day of Transplant

- **Randomisation Form (on day of transplant)**: randomisation date/time, randomisation ID number, treatment arm
- **Pre-transplant blood test results** (Table 4)

Post-Transplant (72 hours)

The 72 hour post-transplant period starts at the time of transplant operation “knife to skin”.

- **Transplant Operation Form**: operation start time, duration, graft placement
- **Fluids**: all fluid input / output, and all patient weight measurements in the first 72 hours post-transplant
- **Blood Products**: all blood products administered in the first 72 hours post-transplant

For peer review only

- **Medications**: all inotropes, diuretics, immunosuppressives, insulin, anti-emetics and electrolyte supplements administered in the first 72 hours post-transplant
- **Symptom Assessment**: daily symptom assessment for nausea, vomiting, headache, pain score, seizures. Pain is graded using an age-appropriate scale (FLACC or Wong-Baker)(24, 25).
- **Blood Pressure**: Maximum and minimum systolic blood pressure sustained on 3 repeated values on each day, for 3 days post-transplant
- **Blood Test Results**: all results to 72 hours post-transplant will be collected (Table 4).

Follow-Up Data Collection

- **Post-Transplant Form**: need for dialysis post-transplant, creatinine at Day 7 post-transplant (+/- 1 day)
- **Hospital Discharge**: date of discharge from hospital

No additional outpatient visits will be required for the research study. Plasma creatinine concentration at Month 3 (+/- 14 days) will be obtained directly from the UKTR held by NHSBT.

Statistical Plan

Sample size

The sample size required is 144 participants randomised. The baseline incidence was determined by analysis of 76 paediatric transplants performed at Great Ormond Street Hospital between 1 January 2015 and 31 March 2018 which showed that 45 children (59%) experienced hyponatraemia within the first 72 hours post-transplant when managed with 0.45% saline 5% glucose fluid(2). The effect size was estimated from the Cochrane systematic review comparing isotonic to hypotonic maintenance fluid in children, in which a 50% reduction in risk of hyponatraemia was shown(11). A greater effect size is anticipated because the rate of fluid delivery to children following kidney transplant significantly exceeds maintenance requirements(9). Using the 50% effect size from the Cochrane review, the assumed incidence of hyponatraemia would be reduced to 29.5%. A two-sided test with 90% power, 5% type I error and 1:1 allocation and allowing for two formal interim analyses for harm or benefit would require 128 participants. After allowing for 10% attrition, for example if a transplant cannot proceed due to a positive cross match, the total number of participants required is 144.

Interim analyses

A group sequential design with O'Brien-Fleming boundaries will inform early stopping of the trial in the case of strong evidence of harm or benefit, while preserving the overall 5% type I error rate. Two interim analyses to test for harm or benefit will be conducted after 70 and 100 participants have been recruited into the trial with primary outcome data. The stopping rules will be used as a guideline, alongside the other safety data available to the DMC, and used as part of their overall assessment of the trial.

Analysis

For peer review only

The analyses will be described in detail in a full Statistical Analysis Plan. This section summarises the main issues.

The population used for efficacy analyses will be a modified intention to treat analysis which will include all randomised participants who receive a transplant, as although unlikely it would be illogical to include any participant who did not receive a transplant after being randomised. This will be the primary analysis for the trial though the primary outcome will also be analysed per protocol.

Characteristics of all randomised patients will be tabulated by arm of the trial to describe the cohort. A CONSORT diagram will be presented to show the flow of patients through the trial.

Primary outcome: proportion of patients who experience hyponatraemia (defined as plasma sodium concentration <135mmol/L) within 72 hours post-transplant. This will be analysed using a logistic regression model adjusting for donor type (living vs deceased donor), patient weight (<20kg vs ≥20kg pre-transplant) and transplant centre as a random effect. Subgroup and sensitivity analyses will be used to assess whether the treatment effect differs according to the fluid regimen used in the standard arm.

Secondary outcomes:
The secondary outcomes which assess the incidence of other electrolyte and acid-base disturbance within 72 hours post transplantation (hyperkalaemia, hypokalaemia, hypernatraemia, acidosis, hypomagnesaemia, hyperglycaemia and excessive rate and magnitude of fall in plasma sodium concentration) will all be analysed using the same model as the primary outcome. Exact logistic regression will be used to compare instances of nausea/vomiting, headache and seizures. Adjusted normal linear regression models will be used to assess differences in the continuous secondary outcomes and an adjusted Cox proportional hazards model will be used to assess differences in time to discharge. The number of changes in intravenous fluid composition within the first 72 hours post-transplant will be summarised by treatment arm.

Any missing primary and secondary outcome data will be summarised. Primary and secondary outcome measures will not be imputed, and these will be treated as missing data and excluded from the relevant analyses. If outcome data are missing for more than 25% of participants, outcomes will not be reported. To explore if missing values have an undue impact on the primary outcome result, a sensitivity analysis using multiple imputation will be performed if the primary outcome is missing in more than 5% of the participants included in the modified intention to treat analysis.

Patient and Public Involvement
A kidney transplant patient and her mother are co-applicants for the grant and members of the Trial Steering Committee (TSC). They make sure that the trial remains patient-centered throughout its delivery and will lead the dissemination of results to patients and families. The research plan was also shaped by a workshop with the Young Persons' Advisory Group at Great Ormond Street Hospital, and involvement of five families with children awaiting kidney transplantation who advised on the burden of intervention.

Ethics, Safety and Dissemination

Ethical Compliance

The Investigator will ensure that this trial is conducted in accordance with the principles of the Declaration of Helsinki, ICH Good Clinical Practice Guidelines and in accordance with the terms and

For peer review only

conditions of the ethical approval given to the trial. A favourable opinion has been given by a Research Ethics Committee (HRA reference 19/LO/1866).

Safety

Adverse Events and Expected Adverse Drug Reactions

Non-serious AEs will not be reported. Plasma-Lyte 148 is a licensed product and therefore adverse reactions which are expected (assessed against the Summary of Product Characteristics(18)) and not serious do not require recording.

SAEs will be notified within 24 hours and monitored regularly by the data monitoring committee (DMC) to ensure ongoing safety of trial participants.

The SAEs listed below are anticipated in this patient group, and have therefore been excluded from trial safety reporting:

1. **Surgical complications:**
    a. Return to theatre for exploration
    b. Haemorrhage
    c. Blood transfusion
    d. Transplant obstruction/dilatation/hydronephrosis
    e. Thrombosis (thrombosis of transplant, DVT, Pulmonary embolus, arteriovenous fistula)
    f. Transplant kidney arteriovenous fistula
    g. Transplant nephrectomy
    h. Haematuria
    i. cystoscopy for evacuation of haematoma or stent removal
    j. renal artery stenosis
    k. bowel injury, obstruction or ischaemia
    l. lymphocele/collection +/-drainage
    m. hernia
    n. urine leak
    o. nephrostomy

2. **Infective complications:**
    a. Viraemia (EBV, CMV, JC and BK viraemia)
    b. Bacteraemia
    c. Fever

3. **Immunological complications:**
    a. Acute rejection
    b. Transplant kidney biopsy

4. **Medication related complications:**
    a. Calcineurin inhibitor nephrotoxicity and neurotoxicity
    b. New onset diabetes

5. **Other:**
    a. Delayed graft function/primary non-function

For peer review only

b. Thrombocytopenia/ thrombotic microangiopathy

Trial oversight

The Trial Management Group (TMG) will be responsible for the day to day running and management of the trial.

The Trial Steering Committee (TSC) will provide overall supervision for the trial and provide advice through its Independent Chair. The ultimate decision on continuation of the trial lies with the TSC.

The Data Monitoring Committee (DMC) will be responsible for safety monitoring. Recommendations made by the DMC to alter the conduct of the study will be forwarded to the TSC for a final decision. The Sponsor will forward such decisions to regulatory authorities, as appropriate. The DMC will be independent of the study team and will have no direct involvement in other aspects of the trial.

Dissemination

The final study data set will be analysed, and results published as soon as possible following completion of study follow-up, final data checks and database lock. The Trial Management Group will form the basis of the Writing Committee and will advise on the nature of publications.

Study findings will be presented to academic and non-academic groups. The patient and public involvement group will play an important part in disseminating the study findings into the public domain. Dissemination to non-academic audiences including service users, commissioners, clinicians and service providers will be facilitated through the use of existing networks e.g. email lists, social media.

All research teams and PPI members involved in the study will be invited to a close out meeting to discuss the findings of the study.

Open access, peer reviewed academic outputs and research reports together with associated summaries and key findings will be produced for funders, policy makers and NHS audiences and held on the study website.

Any publications arising from this study will adhere to the NIHR funding and support outputs guidance.

References

1. Roach JP, Bock ME, Goebel J. Pediatric kidney transplantation. Seminars in pediatric surgery. 2017;26(4):233-40.
2. Hayes W, Longley C, Scanlon N, Bryant W, Stojanovic J, Kessaris N, et al. Plasma electrolyte imbalance in pediatric kidney transplant recipients. Pediatr Transplant. 2019:e13411.
3. Drake K, Nehus E, Goebel J. Hyponatremia, hypo-osmolality, and seizures in children early post-kidney transplant. Pediatr Transplant. 2015;19(7):698-703.
4. Cansick J, Rees L, Koffman G, Van't Hoff W, Bockenhauer D. A fatal case of cerebral oedema with hyponatraemia and massive polyuria after renal transplantation. Pediatr Nephrol. 2009;24(6):1231-4.

For peer review only

5. Arieff AI, Ayus JC, Fraser CL. Hyponatraemia and death or permanent brain damage in healthy children. Bmj. 1992;304(6836):1218-22.
6. Ayus JC, Achinger SG, Arieff A. Brain cell volume regulation in hyponatremia: role of sex, age, vasopressin, and hypoxia. Am J Physiol Renal Physiol. 2008;295(3):F619-24.
7. Gankam Kengne F, Decaux G. Hyponatremia and the Brain. Kidney Int Rep. 2018;3(1):24-35.
8. Giuliani C, Peri A. Effects of Hyponatremia on the Brain. J Clin Med. 2014;3(4):1163-77.
9. Coupe N, O'Brien M, Gibson P, de Lima J. Anesthesia for pediatric renal transplantation with and without epidural analgesia--a review of 7 years experience. Paediatr Anaesth. 2005;15(3):220-8.
10. Forsythe J. Report of the Inquiry into hyponatraemia related deaths. 2018.
11. McNab S, Ware RS, Neville KA, Choong K, Coulthard MG, Duke T, et al. Isotonic versus hypotonic solutions for maintenance intravenous fluid administration in children. Cochrane Database Syst Rev. 2014(12):CD009457.
12. Foster BA, Tom D, Hill V. Hypotonic versus isotonic fluids in hospitalized children: a systematic review and meta-analysis. J Pediatr. 2014;165(1):163-9 e2.
13. Wang J, Xu E, Xiao Y. Isotonic versus hypotonic maintenance IV fluids in hospitalized children: a meta-analysis. Pediatrics. 2014;133(1):105-13.
14. Choong K, Kho ME, Menon K, Bohn D. Hypotonic versus isotonic saline in hospitalised children: a systematic review. Arch Dis Child. 2006;91(10):828-35.
15. McNab S, Duke T, South M, Babl FE, Lee KJ, Arnup SJ, et al. 140 mmol/L of sodium versus 77 mmol/L of sodium in maintenance intravenous fluid therapy for children in hospital (PIMS): a randomised controlled double-blind trial. Lancet. 2015;385(9974):1190-7.
16. Weiss SL, Babl FE, Dalziel SR, Balamuth F. Is chloride worth its salt? Intensive care medicine. 2019;45(2):275-7.
17. National Institute for Health and Care Excellence. Intravenous fluid therapy in children and young people in hospital. 2016.
18. Electronic medicines compendium. Plasma-Lyte 148 (pH 7.4) solution for infusion.
19. Wan S, Roberts MA, Mount P. Normal saline versus lower-chloride solutions for kidney transplantation. Cochrane Database Syst Rev. 2016(8):CD010741.
20. Weinberg L, Harris L, Bellomo R, Ierino FL, Story D, Eastwood G, et al. Effects of intraoperative and early postoperative normal saline or Plasma-Lyte 148(R) on hyperkalaemia in deceased donor renal transplantation: a double-blind randomized trial. British journal of anaesthesia. 2017;119(4):606-15.
21. Adwaney A, Randall DW, Blunden MJ, Prowle JR, Kirwan CJ. Perioperative Plasma-Lyte use reduces the incidence of renal replacement therapy and hyperkalaemia following renal transplantation when compared with 0.9% saline: a retrospective cohort study. Clin Kidney J. 2017;10(6):838-44.
22. Schwartz GJ, Munoz A, Schneider MF, Mak RH, Kaskel F, Warady BA, et al. New equations to estimate GFR in children with CKD. Journal of the American Society of Nephrology : JASN. 2009;20(3):629-37.
23. Lee ST, Cho H. Fluid overload and outcomes in neonates receiving continuous renal replacement therapy. Pediatr Nephrol. 2016;31(11):2145-52.
24. Merkel SI, Voepel-Lewis T, Shayevitz JR, Malviya S. The FLACC: a behavioral scale for scoring postoperative pain in young children. Pediatr Nurs. 1997;23(3):293-7.
25. Garra G, Singer AJ, Taira BR, Chohan J, Cardoz H, Chisena E, et al. Validation of the Wong-Baker FACES Pain Rating Scale in pediatric emergency department patients. Acad Emerg Med. 2010;17(1):50-4.

Acknowledgements
This study is sponsored by Great Ormond Street Hospital for Children NHS Foundation Trust. The study was supported by the Digital Research Environment Team at Great Ormond Street Hospital.

For peer review only

Our thanks to Maya and Sylwia Olejnik-Antkowiak, patient and parent representatives who have contributed to the design of this study, and to the NIHR Great Ormond Street Hospital Young Person's Advisory Group for research (YPAG) for co-designing patient information resources.

Authors' contributions

WNH developed the idea for this study, drafted this manuscript and has oversight of the study with mentorship from MJP. WNH, EL, LP, HT and MJP applied for funding and developed the trial protocol. JW developed the recruitment process evaluation. NK, HHS, WB, AS and SDM are all study co-investigators and contributed to the writing of this protocol. EL, CF and LP are the trial manager, clinical operations manager and trial statistician, respectively, at the NHSBT CTU and contributed to the writing of the protocol and study planning and approval process. All authors read and approved the final manuscript.

Funding statement

This study is funded by the National Institute for Health Research Research for Patient Benefit programme (project reference number NIHR200512). The views expressed are those of the author(s) and not necessarily those of the NIHR or the Department of Health and Social Care.

Competing interests statement

None declared

For peer review only

Table 1: Participating Paediatric Kidney Transplant Centres

Trust	Principal Investigator
Royal Belfast Hospital for Sick Children	Dr Mairead Convery
Birmingham Childrens Hospital	Dr Mordi Mourah
Bristol Royal Hospital for Children	Dr Jan Dudley
Great Ormond Street Hospital	Dr Wesley Hayes
Evelina London Children's Hospital	Dr Nick Ware
Leeds Children's Hospital	Dr Pallavi Yadav
Royal Manchester Children's Hospital	Dr Mohan Shenoy
Great North Children's Hospital	Dr Michal Malina
Nottingham Children's Hospital	Dr Martin Christian
University Hospital of Wales*	Dr Shivaram Hegde
University Hospital Southampton*	Dr Shuman Haq
Alder Hey Children's Hospital*	Dr Henry Morgan

* Participant identification centre

For peer review only

Table 2: Electrolyte composition of PlasmaLyte-148 and commonly used Standard Care fluids

	Na+ (mmol/l)	K+ (mmol/l)	Mg2+ (mmol/l)	Cl- (mmol/l)	Acetate (mmol/l)	Gluconate (mmol/l)
PlasmaLyte-148	140	5	1.5	98	27	23
0.9% saline	154			154		
0.45% saline 5% glucose	77			77		

For peer review only

Table 3: Schedule of Procedures

No additional tests are required for PLUTO trial participants. This table summarises the data required at each timepoint:

	Screening	Baseline (Pre-Tx)	At Tx	Day 1 Post-Tx	Day 2 Post-Tx	Day 3 Post-Tx	Day 7 Post-Tx	Month 3 Post-Tx	Hospital Discharge
**ENROLMENT**									
Qualitative Sub-Study – give Q1	X								
Eligibility assessment	X								
Informed consent		X							
Baseline Characteristics		X							
Re-confirm consent/assent			X						
Randomisation			X						
Qualitative Sub-Study – give Q2							X		
**TREATMENT**									
Administration of trial fluid			X	X	X	X			
**ASSESSMENTS**									
Transplant Operation Data			X						
Blood Results**		X	All results for 72 hours from transplant 'knife-to-skin'						
Blood Gas Results**		X	All results for 72 hours from transplant 'knife-to-skin'						
Patient Weight		X	All weights for 72 hours from transplant 'knife-to-skin'						
Fluid Data			All fluid for 72 hours from transplant 'knife-to-skin'						
Medication Data			All meds* for 72 hours from transplant 'knife-to-skin'						
Symptom Assessment				X	X	X			
Dialysis				X	X	X			
Blood Pressure		X		X	X	X			
Safety Reporting			X	X	X	X			
Transplant Graft Function							X	X***	
Discharge Date									X

For peer review only

*Data must be collected on all inotropes, diuretics, immunosuppressives, insulin, anti-emetics and electrolyte supplements administered during this 72 hour period.
**Blood Results and Venus Blood Gas data will be obtained directly from the Trust Pathology System (*where possible*), anonymised and then transferred to the Digital Research Environment at GOSH.
***Data will be obtained directly from the UKTR, held by NHSBT(e.g. patient ethnicity, blood group, donor details, matching details, transplant graft function).

Table 4. Blood parameters

Test	Preferred Units
Plasma sodium level	mmol/L
Plasma potassium level	mmol/L
Plasma urea level	mmol/L
Plasma creatinine level	micromol/L
Plasma chloride level	mmol/L
Plasma bicarbonate level	mmol/L
Plasma magnesium level	mmol/L
Plasma calcium level	mmol/L
Plasma inorganic phosphate level	mmol/L
Blood glucose level	mmol/L
Haemoglobin mass concentration in blood	g/L
Blood gas pH	-
Blood gas – bicarbonate	mmol/L
Blood gas – chloride	mmol/L
Blood gas – sodium	mmol/L
Blood gas – potassium	mmol/L
Blood gas - ionised calcium	mmol/L
Blood gas – glucose	mmol/l

Figure Legends
Figure 1 – PLUTO trial flowchart

Figure 2: Qualitative Sub-Study Questionnaires

For peer review only

Figure 1 – PLUTO trial flowchart

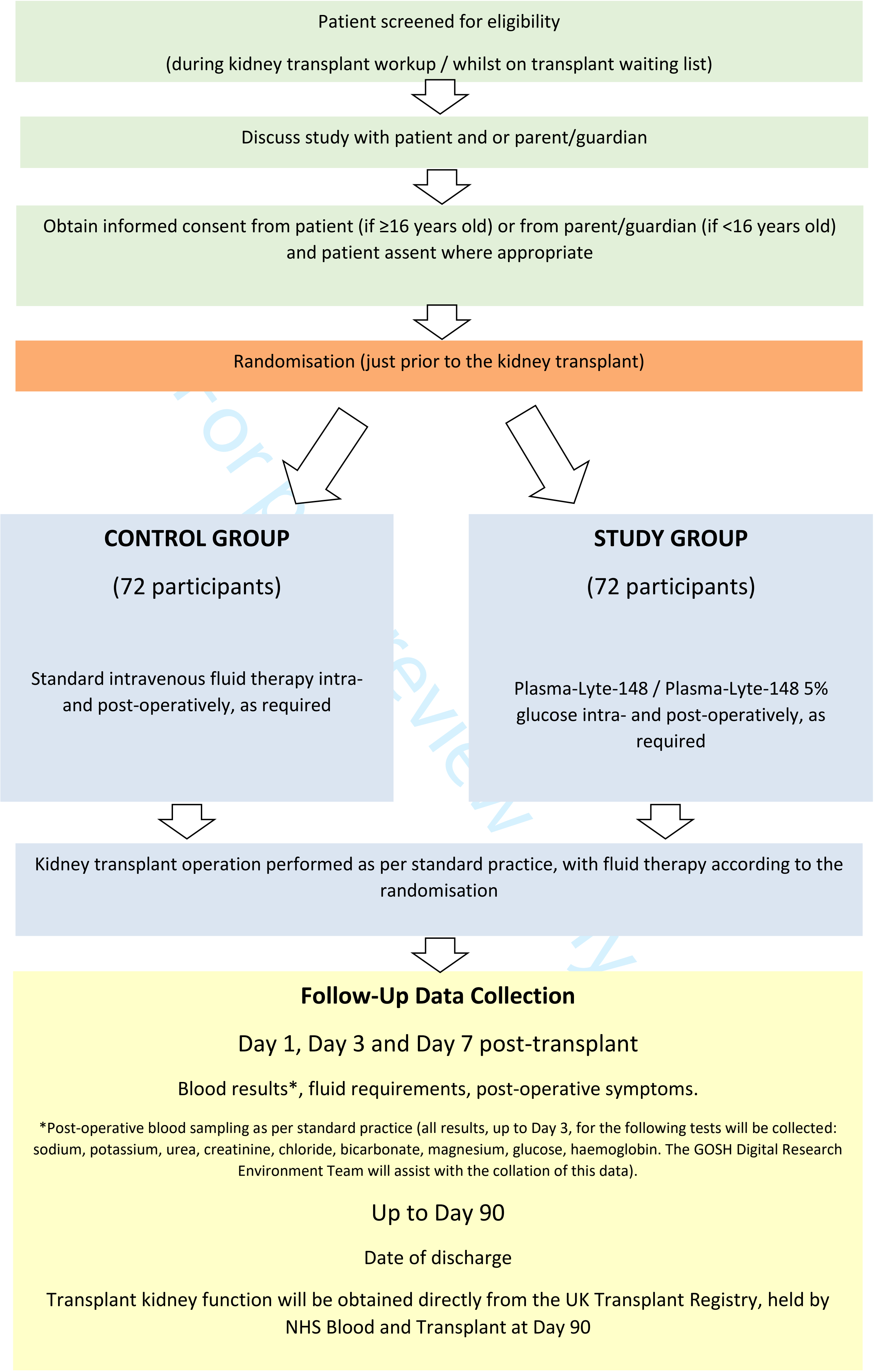

For peer review only - http://bmjopen.bmj.com/site/about/guidelines.xhtml

Figure 2: Qualitative Sub-Study Questionnaires

QUESTIONNAIRE 1 – BEFORE THE TRANSPLANT

	Strongly agree	Agree	Neither agree nor disagree	Disagree	Strongly disagree
I was satisfied with how I was **approached** about PLUTO	◯	◯	◯	◯	◯
I understood the **information** that I received from the doctor/research nurse about PLUTO	◯	◯	◯	◯	◯
I had sufficient opportunity to ask **questions** about PLUTO	◯	◯	◯	◯	◯
Any questions I had about PLUTO were answered in a way that I could **understand**	◯	◯	◯	◯	◯
I was satisfied with the **consent** process for PLUTO	◯	◯	◯	◯	◯
I had enough **time** to think about whether or not to consent to my child participating in PLUTO	◯	◯	◯	◯	◯

QUESTIONNAIRE 2 – AFTER THE TRANSPLANT

	Strongly agree	Agree	Neither agree nor disagree	Disagree	Strongly disagree
I was satisfied with how I was **approached** about PLUTO on the night of transplant	◯	◯	◯	◯	◯
I remembered the **information** I had previously been given about PLUTO	◯	◯	◯	◯	◯
I had sufficient opportunity to ask **questions** about PLUTO on the night of transplant	◯	◯	◯	◯	◯
Any questions I had about PLUTO were answered in a way that I could **understand**	◯	◯	◯	◯	◯
I was satisfied with the **re-consent** process for PLUTO on the night of transplant	◯	◯	◯	◯	◯
I was happy with my original **decision** to consent to my child participating in PLUTO	◯	◯	◯	◯	◯

For peer review only

SPIRIT
STANDARD PROTOCOL ITEMS: RECOMMENDATIONS FOR INTERVENTIONAL TRIALS

SPIRIT 2013 Checklist: Recommended items to address in a clinical trial protocol and related documents*

Section/item	Item No	Description	Addressed on page number
**Administrative information**			
Title	1	Descriptive title identifying the study design, population, interventions, and, if applicable, trial acronym	_____1________
Trial registration	2a	Trial identifier and registry name. If not yet registered, name of intended registry	_____ 2_______
	2b	All items from the World Health Organization Trial Registration Data Set	______2_______
Protocol version	3	Date and version identifier	Manuscript based on full trial protocol v2.0 dated 23 March 2021
Funding	4	Sources and types of financial, material, and other support	______13_______
Roles and responsibilities	5a	Names, affiliations, and roles of protocol contributors	________1_____
	5b	Name and contact information for the trial sponsor	_______13______
	5c	Role of study sponsor and funders, if any, in study design; collection, management, analysis, and interpretation of data; writing of the report; and the decision to submit the report for publication, including whether they will have ultimate authority over any of these activities	_______13_____

For peer review only

	5d	Composition, roles, and responsibilities of the coordinating centre, steering committee, endpoint adjudication committee, data management team, and other individuals or groups overseeing the trial, if applicable (see Item 21a for data monitoring committee)	______11_______
**Introduction**			
Background and rationale	6a	Description of research question and justification for undertaking the trial, including summary of relevant studies (published and unpublished) examining benefits and harms for each intervention	____4&5_____
	6b	Explanation for choice of comparators	_____5_________
Objectives	7	Specific objectives or hypotheses	______5 (aim)__
Trial design	8	Description of trial design including type of trial (eg, parallel group, crossover, factorial, single group), allocation ratio, and framework (eg, superiority, equivalence, noninferiority, exploratory)	_______5______
**Methods: Participants, interventions, and outcomes**			
Study setting	9	Description of study settings (eg, community clinic, academic hospital) and list of countries where data will be collected. Reference to where list of study sites can be obtained	_______5_____
Eligibility criteria	10	Inclusion and exclusion criteria for participants. If applicable, eligibility criteria for study centres and individuals who will perform the interventions (eg, surgeons, psychotherapists)	______6_______
Interventions	11a	Interventions for each group with sufficient detail to allow replication, including how and when they will be administered	______7_____
	11b	Criteria for discontinuing or modifying allocated interventions for a given trial participant (eg, drug dose change in response to harms, participant request, or improving/worsening disease)	_____7_____
	11c	Strategies to improve adherence to intervention protocols, and any procedures for monitoring adherence (eg, drug tablet return, laboratory tests)	N/A, intervention is delivered by clinical staff

For peer review only - http://bmjopen.bmj.com/site/about/guidelines.xhtml

	11d	Relevant concomitant care and interventions that are permitted or prohibited during the trial	_____7_____
Outcomes	12	Primary, secondary, and other outcomes, including the specific measurement variable (eg, systolic blood pressure), analysis metric (eg, change from baseline, final value, time to event), method of aggregation (eg, median, proportion), and time point for each outcome. Explanation of the clinical relevance of chosen efficacy and harm outcomes is strongly recommended	_____5&6_____
Participant timeline	13	Time schedule of enrolment, interventions (including any run-ins and washouts), assessments, and visits for participants. A schematic diagram is highly recommended (see Figure)	_____15________
Sample size	14	Estimated number of participants needed to achieve study objectives and how it was determined, including clinical and statistical assumptions supporting any sample size calculations	______8_______
Recruitment	15	Strategies for achieving adequate participant enrolment to reach target sample size	_______7______
**Methods: Assignment of interventions (for controlled trials)**			
Allocation:			
Sequence generation	16a	Method of generating the allocation sequence (eg, computer-generated random numbers), and list of any factors for stratification. To reduce predictability of a random sequence, details of any planned restriction (eg, blocking) should be provided in a separate document that is unavailable to those who enrol participants or assign interventions	_______6______
Allocation concealment mechanism	16b	Mechanism of implementing the allocation sequence (eg, central telephone; sequentially numbered, opaque, sealed envelopes), describing any steps to conceal the sequence until interventions are assigned	_______6______
Implementation	16c	Who will generate the allocation sequence, who will enrol participants, and who will assign participants to interventions	_______6______
Blinding (masking)	17a	Who will be blinded after assignment to interventions (eg, trial participants, care providers, outcome assessors, data analysts), and how	N/A – open label trial
	17b	If blinded, circumstances under which unblinding is permissible, and procedure for revealing a participant's allocated intervention during the trial	____N/A_____

Methods: Data collection, management, and analysis

Data collection methods	18a	Plans for assessment and collection of outcome, baseline, and other trial data, including any related processes to promote data quality (eg, duplicate measurements, training of assessors) and a description of study instruments (eg, questionnaires, laboratory tests) along with their reliability and validity, if known. Reference to where data collection forms can be found, if not in the protocol	______7_______
	18b	Plans to promote participant retention and complete follow-up, including list of any outcome data to be collected for participants who discontinue or deviate from intervention protocols	N/A, data collection only during hospitalisation, follow up data will be obtained from a patient registry
Data management	19	Plans for data entry, coding, security, and storage, including any related processes to promote data quality (eg, double data entry; range checks for data values). Reference to where details of data management procedures can be found, if not in the protocol	More details are included in the study's data management and statistical analysis plan - limited by word count in the manuscript
Statistical methods	20a	Statistical methods for analysing primary and secondary outcomes. Reference to where other details of the statistical analysis plan can be found, if not in the protocol	______9_______
	20b	Methods for any additional analyses (eg, subgroup and adjusted analyses)	______9_______
	20c	Definition of analysis population relating to protocol non-adherence (eg, as randomised analysis), and any statistical methods to handle missing data (eg, multiple imputation)	______9_______

Methods: Monitoring

Data monitoring	21a	Composition of data monitoring committee (DMC); summary of its role and reporting structure; statement of whether it is independent from the sponsor and competing interests; and reference to where further details about its charter can be found, if not in the protocol. Alternatively, an explanation of why a DMC is not needed	______11_______
	21b	Description of any interim analyses and stopping guidelines, including who will have access to these interim results and make the final decision to terminate the trial	_____9______
Harms	22	Plans for collecting, assessing, reporting, and managing solicited and spontaneously reported adverse events and other unintended effects of trial interventions or trial conduct	____10&11___
Auditing	23	Frequency and procedures for auditing trial conduct, if any, and whether the process will be independent from investigators and the sponsor	_____N/A____
**Ethics and dissemination**			
Research ethics approval	24	Plans for seeking research ethics committee/institutional review board (REC/IRB) approval	______2_______
Protocol amendments	25	Plans for communicating important protocol modifications (eg, changes to eligibility criteria, outcomes, analyses) to relevant parties (eg, investigators, REC/IRBs, trial participants, trial registries, journals, regulators)	______11______
Consent or assent	26a	Who will obtain informed consent or assent from potential trial participants or authorised surrogates, and how (see Item 32)	______6_______
	26b	Additional consent provisions for collection and use of participant data and biological specimens in ancillary studies, if applicable	___N/A______
Confidentiality	27	How personal information about potential and enrolled participants will be collected, shared, and maintained in order to protect confidentiality before, during, and after the trial	Available in the protocol and patient documents, - limited by word count in the manuscript

peer review only

Declaration of interests	28	Financial and other competing interests for principal investigators for the overall trial and each study site	______13_______
Access to data	29	Statement of who will have access to the final trial dataset, and disclosure of contractual agreements that limit such access for investigators	Details available on the ISRCTN registry – limited by word count in the manuscript
Ancillary and post-trial care	30	Provisions, if any, for ancillary and post-trial care, and for compensation to those who suffer harm from trial participation	Details included in the full trial protocol - limited by word count in the manuscript
Dissemination policy	31a	Plans for investigators and sponsor to communicate trial results to participants, healthcare professionals, the public, and other relevant groups (eg, via publication, reporting in results databases, or other data sharing arrangements), including any publication restrictions	______2_____
	31b	Authorship eligibility guidelines and any intended use of professional writers	_____N/A______
	31c	Plans, if any, for granting public access to the full protocol, participant-level dataset, and statistical code	_____N/A______
**Appendices**			
Informed consent materials	32	Model consent form and other related documentation given to participants and authorised surrogates	__Attached ___
Biological specimens	33	Plans for collection, laboratory evaluation, and storage of biological specimens for genetic or molecular analysis in the current trial and for future use in ancillary studies, if applicable	___N/A______

*It is strongly recommended that this checklist be read in conjunction with the SPIRIT 2013 Explanation & Elaboration for important clarification on the items. Amendments to the protocol should be tracked and dated. The SPIRIT checklist is copyrighted by the SPIRIT Group under the Creative Commons "Attribution-NonCommercial-NoDerivs 3.0 Unported" license.

For peer review only - http://bmjopen.bmj.com/site/about/guidelines.xhtml
